# Spatially nanoconfined N-type polymer semiconductors for stretchable ultrasensitive X-ray detection

**DOI:** 10.1038/s41467-022-34968-1

**Published:** 2022-11-22

**Authors:** Yangshuang Bian, Kai Liu, Yang Ran, Yi Li, Yuanhong Gao, Zhiyuan Zhao, Mingchao Shao, Yanwei Liu, Junhua Kuang, Zhiheng Zhu, Mingcong Qin, Zhichao Pan, Mingliang Zhu, Chenyu Wang, Hu Chen, Jia Li, Xifeng Li, Yunqi Liu, Yunlong Guo

**Affiliations:** 1 Beijing National Laboratory for Molecular Sciences, Key Laboratory of Organic Solids, Institute of Chemistry Chinese Academy of Sciences, Beijing, 100190 China; 2 School of Chemical Sciences, University of Chinese Academy of Sciences, Beijing, 100049 China; 3 Key Laboratory of Advanced Display and System Applications of Ministry of Education, Shanghai University, Shanghai, 200072 China; 4 School of Advanced Materials, Shenzhen Graduate School, Peking University, Shenzhen, 518055 China; 5 College of Engineering Physics, Shenzhen Technology University, Shenzhen, 518118 China

**Keywords:** Electronic devices, Conjugated polymers

## Abstract

Polymer semiconductors are promising candidates for wearable and skin-like X-ray detectors due to their scalable manufacturing, adjustable molecular structures and intrinsic flexibility. Herein, we fabricated an intrinsically stretchable n-type polymer semiconductor through spatial nanoconfinement effect for ultrasensitive X-ray detectors. The design of high-orientation nanofiber structures and dense interpenetrating polymer networks enhanced the electron-transporting efficiency and stability of the polymer semiconductors. The resultant polymer semiconductors exhibited an ultrahigh sensitivity of 1.52 × 10^4^ μC Gy_air_^−1^ cm^−2^, an ultralow detection limit of 37.7 nGy_air_ s^−1^ (comparable to the record-low value of perovskite single crystals), and polymer film X-ray imaging was achieved at a low dose rate of 3.65 μGy_air_ s^−1^ (about 1/12 dose rate of the commercial medical chest X-ray diagnosis). Meanwhile, the hybrid semiconductor films could sustain 100% biaxial stretching strain with minimal degeneracy in photoelectrical performances. These results provide insights into future high-performance, low-cost e-skin photoelectronic detectors and imaging.

## Abstract (web summary)

Wearable and skin-like X-ray detectors need intrinsic flexibility and high sensitivity. Here Guo et al. develop a stretchable n-type polymer semiconductor with aligned nanofiber networks which achieve photosensitivity of 1.52×10^4 μC Gyair^-1 cm^−2 and low detection limit of 37.7 nGyair s^-1.

## Introduction

The continuous quest for portable inspection equipment and implantable healthcare monitors in recent years is facilitating the development of next-generation flat-panel detectors. Actually, X-ray detectors with ultra-flexibility, high skin conformability and superior spatial resolution are easily integrated with curved objects, moving entities, and internal physiological systems, and they demonstrate tremendous potentials to shape human lives for more convenience and safety^1,2^. Consequently, the current focus is mainly on increasing X-ray detection sensitivities by exploring innovative materials (such as perovskites^3–7^, metal–organic frameworks^8,9^, organic small molecules^10–13^, and organic–inorganic hybrid materials^14–17^) and designing device configurations (such as heterojunction-based X-ray phototransistors^18,19^), with the aim of significantly enhancing the conversion efficiencies of X-ray detectors and simplifying the manufacturing procedures. However, none of the aforementioned materials can be adapted into skin-like photoelectric devices due to their stiffness and incompatibility with the complex surface of organisms. Thus, the development of intrinsically flexible and highly sensitive materials for skin-like X-ray detection is of high importance.

Polymer semiconductors are characterized by designable molecular structure, intrinsic flexibility, solution processability, and cost-effective advantages, which have since been extensively employed in flexible and stretchable electronic devices^20–22^. Currently developed hybrid polymer semiconductors consisting of elastomers and conjugated polymers have been certified to sustain high biaxial stretchability up to 100% strain with minimal degradation of electrical performances^23–27^. Nonetheless, only a few p-type conjugated polymers, such as poly(3-hexylthiophene) and diketopyrrolopyrrole-based polymers^24,28,29^, and almost no n-type polymers were reported for skin-like devices. Actually, a large number of n-type conjugated polymers have been invented for photoelectronic devices, such as organic solar cells, organic light-emitting devices, and complementary logic circuitry^30^. However, the non-existing methods for tuning tensile properties as well as the poor stability of n-type conjugated polymers pose a huge challenge in achieving both stretchability and efficient electron transport for the materials. Another challenge lies in the n-type photoactive materials that are still developing, as they are required to display high sensitivities, fast responses, and good stabilities even under strain and irradiation for intrinsically stretchable detectors.

**Fig. 1:**
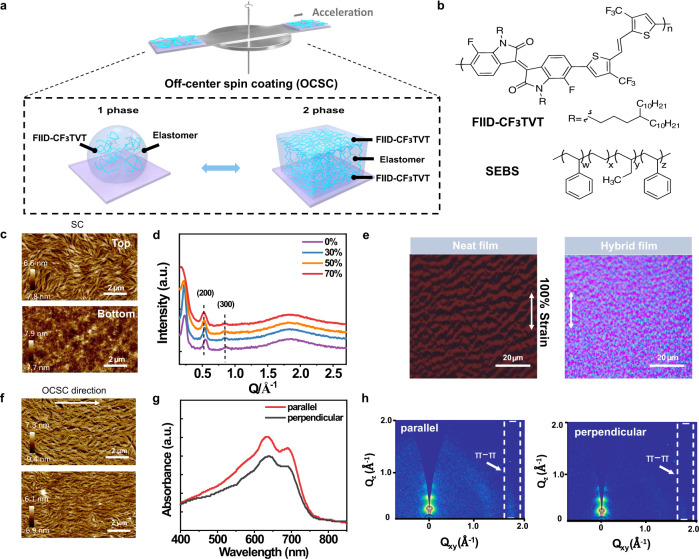
Achieving highly aligned interpenetrating polymer fiber networks of stretchable n-type hybrid polymer semiconductor films through OCSC method combined with the spatial nanoconfinement effect. **a** Three-dimensional schematic representation of the spatially nanoconfined polymer film with the highly aligned interpenetrating polymer fiber networks through the OCSC method. **b** Chemical structures of the polymer semiconductor FIID-CF_3_TVT and SEBS elastomer. **c** AFM height images of the top and bottom surfaces of the hybrid polymer semiconductor films with 70 wt% SEBS through spin-coating (SC) method. **d** GIXRD line cuts for the polymer semiconductor films with different SEBS contents along the **Q**_xy_ axes (The lines cuts are offset for clarity). **e** Optical microscope images of the neat film (left) and the optimized hybrid polymer semiconductor film (right) under 100% strain. **f** AFM height images of the top and bottom surfaces of the hybrid polymer semiconductor film with 70 wt% SEBS through OCSC method (off-center distance = 2 cm). **g** Absorption spectra of polarized ultraviolet–visible spectroscopy from the OCSC hybrid polymer semiconductor film parallel and perpendicular to the radial coating direction. **h** GIWAXS images of the OCSC hybrid polymer semiconductor film, with the incident beam orientation parallel (left) and perpendicular (right) to the coating direction. The dashed boxes highlight the in-plane π–π stacking peaks.

Based on these existing problems and challenges, we fabricated the intrinsically stretchable n-type hybrid polymer semiconductor through spatial nanoconfinement effect using a nonchlorinated solvent-processed polymer (FIID-CF_3_TVT)^31^ as both the photosensitive and electron-transporting material. The highly aligned interpenetrating polymer nanofiber networks and the low trap density of the FIID-CF_3_TVT-based hybrid polymer semiconductor significantly improved the electron-transporting efficiency by the off-center spin-coating (OCSC) methodology, as illustrated in Fig. 1a. The existence of fluorine atoms was beneficial for decreasing the susceptibility of the polymer semiconductor to moisture, oxygen, and high-energy photon irradiation^32^. The prepared stretchable n-type hybrid polymer semiconductor showed ultrahigh photosensitivity (1.52 × 10^4^ μC Gy_air_^−1^ cm^−2^, which is one of the highest values reported for X-ray detectors), an ultralow limit of detection (37.7 nGy_air_ s^−1^, that is comparable to the existing record-low value reported for CH_3_NH_3_PbBr_3-x_Cl_x_ perovskite single crystals^33^), and excellent mechanical stability (>1000 stretching cycles under 25% strain). The enhanced electron transport, especially the subthreshold swing that was significantly reduced by six times, facilitated a fast response time of 40 ms (which was nearly 1000 times quicker than the reported organic X-ray detectors based on OFETs^10^) and a high on-current/off-current (*I*_on_/*I*_off_) ratio of 10^2^ (that is 25 times higher than the reported organic semiconductor of TIPS-pentacene^12^). In this work, the polymer X-ray imaging was achieved at a low-dose rate of 3.65 μGy_air_ s^−1^, which will afford insights toward developing high-performance and low-cost imaging systems.

## Results

### Control of spatially nanoconfined morphology and realization of high-orientation nanofibers

In this stretchable hybrid semiconducting system, an n-type polymer semiconductor (FIID-CF_3_TVT)^31^ was employed as the electron-transporting component while an elastomer, styrene-ethylene-butylene styrene elastomer (SEBS), was used as the plasticizing component (the chemical structures of FIID-CF_3_TVT and SEBS are shown in Fig. 1b). The similar surface energies of the conjugated polymers and elastomer polymers ensured the formation of a homogeneous microphase separation and spatially nanoconfined morphology in the hybrid system^23^ (Supplementary Fig. 1 and Supplementary Table 1), which enabled high strain-tolerance capacity of the conjugated polymers without sacrificing its intrinsic photoelectronic properties^34^. However, the influencing factors on the formation of the spatially nanoconfined morphology in a conjugated polymer/elastomer hybrid system remain unclarified. To address this conundrum, we explored the spatially nanoconfined morphology of the n-type hybrid polymer semiconductor films by altering several parameters including the SEBS contents, solvent type, annealing temperature, and spin-coating conditions. From atomic force microscopy (AFM) height images, the nanofibers of the hybrid polymer semiconductor films were thinner and denser with increasing SEBS content and spin-coating speed, which might result from the slow nucleation and suppressed crystal growth of the conjugated polymer semiconductor wrapped by the SEBS matrix (Supplementary Figs. 2 and 3). Nonetheless, the variation of the annealing temperatures showed minimal effects on the nanofiber morphologies (Supplementary Fig. 2). The solvents produced certain effects on the polymer networks. For example, the hybrid polymer semiconductor films with 50 wt% SEBS prepared from chlorobenzene exhibited a denser network than that from the dimethylbenzene. Therefore, these results provide a basis for the rational predictions and designs of the spatial nanoconfined morphologies of hybrid polymer semiconducting systems.

To obtain a hybrid polymer semiconductor with desirable nanoconfined morphology, the preparation conditions were optimized by adjusting the blending ratio of FIID-CF_3_TVT and SEBS to 3:7, as well as adopting a solution concentration of 10 mg mL^−1^, spin-coating speed of 3000 rpm and annealing temperature of 220 °C (Fig. 1c). An in-depth elemental analysis by X-ray photoelectron spectroscopy (XPS) showed the formation of a microphase-separated nanoconfined structure in the hybrid polymer semiconductor (Supplementary Fig. 4a). Combined with the top and bottom surface morphologies by AFM examination (Fig. 1c), it was confirmed that the FIID-CF_3_TVT rich-phase mainly occupied the top and bottom surfaces, whereas the interlayer was dominated by the elastic SEBS phase in the optimized film with 70 wt% SEBS. As observed by ultraviolet/visible (UV–Vis) spectroscopy measurements (Supplementary Fig. 4b), the absorption peaks of the hybrid polymer semiconductor films demonstrated a significant redshift compared with the neat FIID-CF_3_TVT films, which revealed an increased aggregation in short range. A slight leftward shift of (h00) diffraction peak was observed by grazing incidence X-ray diffraction (GIXRD), indicating that the introduction of SEBS was beneficial for the improvement of the lamellar stacking distance with little reduction in crystallinity (Fig. 1d and Supplementary Fig. 5). The stretchability of the semiconductor films was then investigated by employing sequential thermal lamination-transfer processes and in-situ stretching approaches (Supplementary Fig. 6). Unlike neat polymer semiconductor films with obvious cracks only under 50% strain, the hybrid polymer semiconductor films could be stretched to 100% strain without obvious crack formation (Fig. 1e and Supplementary Fig. 7). Consequently, the controlled spatial nanoconfined morphology and the designed interpenetrating polymer nanofiber network collectively ensured a high stretchability and high electron-transporting efficiency of the hybrid polymer semiconductor system.

The electrical performances of the n-type hybrid polymer semiconductor were explored based on the organic field-effect transistors (OFETs) with a bottom-gate top-contact (BGTC) configuration on Si/SiO_2_ substrates (Supplementary Fig. 8a). The mobility (*µ*), threshold voltage (*V*_th_) and subthreshold swing (SS) are important parameters for evaluating the electronic characteristics of OFETs. Compared with neat FIID-CF_3_TVT, the electron mobilities of the hybrid polymer semiconductors with 70 wt% SEBS slightly decreased from 0.47 to 0.31 cm^2^ V^−1^ s^−1^ as the threshold voltage decreased even with a higher current (Supplementary Figs. 8 and 9). However, the subthreshold swing and threshold voltage were significantly reduced by about 6 times (from 6 to 1 V/dec for SS and 30 to 5 V for *V*_th_), respectively. Therefore, the OFETs with hybrid polymer semiconductors significantly improved overall field-effect properties. This mainly accounts for the reason why the formation of the spatially nanoconfined morphology ensured better interfacial contact between the semiconductor layer and the source/drain electrodes, which was beneficial for the decrease of contact resistance and interface defects.

Previous works reported that the formation of highly aligned polymer molecular chains effectively improved carrier-transporting efficiencies^35–38^. Furthermore, a well-aligned interpenetrating polymer nanofiber network in the hybrid polymer semiconductor films based on the OCSC method was designed (Fig. 1f), and the normalized polarized-absorption spectra were conducted to characterize the highly aligned semiconductor films. Compared with the hybrid polymer semiconductor films obtained by the conventional spin-coating method, the above-mentioned film prepared through the OCSC method exhibited obvious anisotropic light absorption with stronger absorption intensity when the polarized light was parallel to the orientation direction than perpendicular to the direction due to the nanofiber alignment effect (Fig. 1g). To further probe the molecular chain alignment of the conjugated polymers in the hybrid semiconductor system, the grazing incidence X-ray diffraction wide-angle X-ray scattering (GIWAXS) images with different incident X-ray directions were measured (Fig. 1h). The resultant hybrid polymer semiconductor films obtained based on the OCSC method displayed a strong π−π stacking diffraction peak along the polymer molecular orientation, whereas that in the perpendicular direction was negligible (Fig. 1h), illustrating the edge-on orientational formation of crystalline domains^35^. By contrast, the hybrid polymer semiconductor obtained based on the spin-coating method showed no dependence on the polarized light direction and the incident X-ray directions (Supplementary Fig. 10).

### Electrical performance of stretchable n-type transistors

**Fig. 2:**
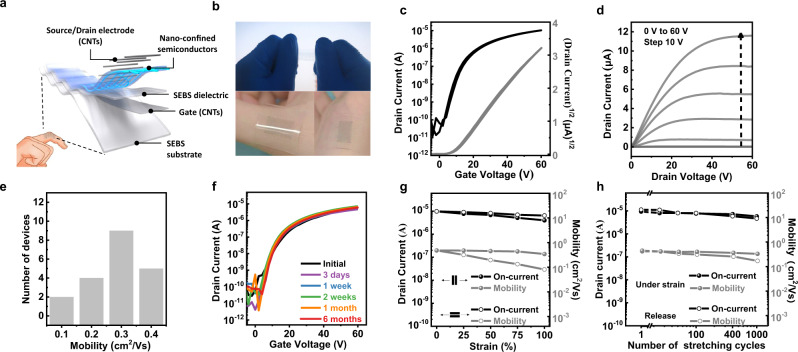
Intrinsically stretchable transistors fabricated using the OCSC n-type hybrid polymer semiconductor films. **a** Device structure of the STOFETs with channel length/width of 200 µm/4 mm and dielectric thickness of about 2000 nm. **b** Images showing the skin-like characteristics of the stretchable transistor. **c** A typical transfer curve (*V*_DS_ = 60 V, with *V*_DS_ representing drain voltage) from the STOFET device fabricated using the OCSC n-type hybrid polymer semiconductor films in its initial condition. **d** A typical output characteristic obtained from the OCSC n-type hybrid polymer semiconductor films. **e** Distribution of the carrier mobility from 20 devices in a fully stretchable transistor array. **f** The real ‘working lifetime‘ of the STOFETs with the n-type hybrid semiconductor film stored for ~6 month in a nitrogen-filled glove box without any encapsulation. **g** Changes in the on current and mobility [calculated with measured geometry and dielectric capacitance under strain (Supplementary Table 2 and Supplementary Fig. 18); also for the values in **h**] with strains up to 100%, both parallel to and perpendicular to the charge transport direction. **h** Changes in the STOFET characteristics (including on current and mobility) after multiple stretching-releasing cycles (up to 1000 cycles) at 25% strain along the charge-transporting direction.

The intrinsically stretchable organic field-effect transistors (STOFETs) with BGTC configuration were fabricated using the optimized hybrid polymer semiconductor (Fig. 2a and Supplementary Fig. 11). These STOFETs consisted of CNTs as the gate, source, and drain electrodes, the SEBS elastomer as the dielectric and substrate, and the hybrid polymer semiconductor as the active layer. Notably, the bottom surface of the hybrid polymer semiconductor films showed more obvious nanoconfined morphologies than the top surface, which favored the fabrication of STOFETs through soft contact lamination and transfer methods (Fig. 1c, f). The STOFETs displayed high transparency and strong adhesion for smart wearable electronic epidermis (Fig. 2b). As shown from the transfer and output characteristics of the STOFETs, a maximum carrier mobility of 0.49 cm^2^ V^−1^ s^−1^, a high on-current/off-current (*I*_on_/*I*_off_) ratio of about 8 × 10^5^ and a low threshold voltage of ~5 V were realized (Fig. 2c, d). Moreover, the average carrier mobility of the STOFETs was 0.38 cm^2^ V^−1^ s^−1^, which is approximately two-fold higher than their spin-coating counterparts (Fig. 2e and Supplementary Fig. 12). It is universally acknowledged that n-type conjugated polymers are very susceptible to air and moisture, thus showing inferior environmental stability compared with p-type conjugated polymers. To evaluate the real ‘working lifetime’, the prepared STOFETs were measured after storage in a nitrogen-filled glove box for a long time. Fortunately, there is no noticeable degradation in their electrical properties after 6 months. This is attributed to the protection of the nanoconfined polymer fibers by the wrapped SEBS (Fig. 2f). Even after storage in air for one week, the hybrid polymer semiconductors still exhibited desirable field-effect characteristics unlike the comparative neat semiconductors with the severely degraded on-current by ~2 orders of magnitude after three days in the same environment (Supplementary Fig. 13). The improved environmental stability of the hybrid polymer semiconductor was primarily due to the metastable effect induced by the long-term chain relaxation in the spatially nanoconfined structure. Meanwhile, the good stability of the nanoconfined films was supported by the unchanged film morphology of the hybrid polymer semiconductor during storage for 6 months (Supplementary Fig. 14).

The strain-tolerance capacity of stretchable electronic devices is of great significance in practical applications. It is observed from the mechanical-electrical relationship that the prepared STOFETs achieved relatively stable field-effect characteristics (including mobility, *I*_on_/*I*_off_ and *V*_th_) even when subjected to 100% biaxial stretching strains (Fig. 2g and Supplementary Fig. 15). To investigate the cycling stability, the STOFETs were subjected to 0–1000 stretching-releasing cycles at 25% strain along the electron-transporting direction. The 25% stretching strain could satisfy most of the practical applications for wearable electronic devices^23^. During the stretching-releasing cycles, there was a minimal degradation in the on current and carrier mobility from 0.49 to 0.32 cm^2^ V^−1^ s^−1^ (Fig. 2h and Supplementary Fig. 15d). In comparison, the electrical performance of the neat semiconductor film decreased rapidly before being eventually exterminated under 20% strain on account of the formation of severe cracks (Supplementary Figs. 7 and 16). The improved stretching stability of the STOFETs with the hybrid polymer semiconductor is accounted for the change in film morphology as depicted in Supplementary Fig. 17. The nanoconfined hybrid polymer semiconductor film showed no obvious crack formation after repeated stretching-releasing cycles at 25% strain, except for the slightly increased roughness. However, when subjected to similar stretching strains and cycles, there were visible and numerous large cracks with an average width of ~200 nm (Supplementary Fig. 17) on the neat polymer semiconductor films. Consequently, the formation of the spatially nanoconfined structure significantly improved the mechanical stretchability and electron-transporting efficiency of the n-type hybrid polymer semiconductor.

### Response of stretchable X-ray detectors to X-ray irradiation

**Fig. 3:**
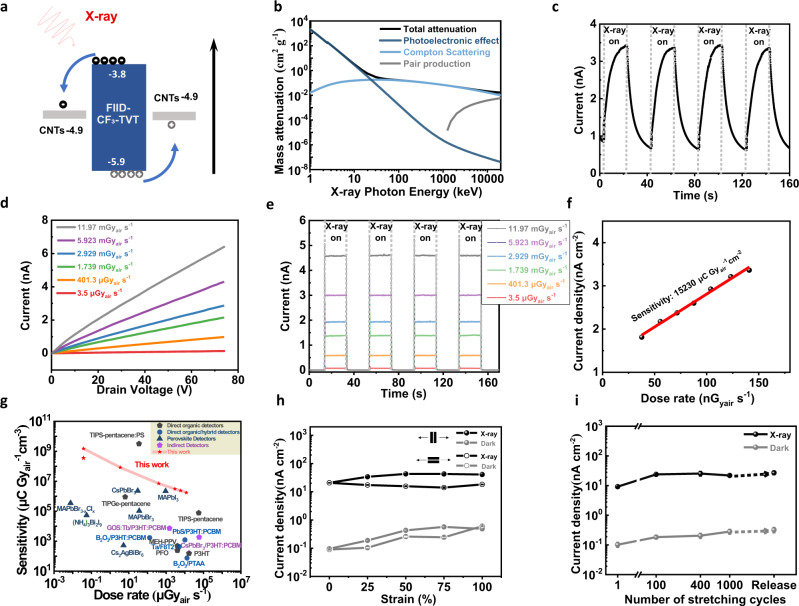
Intrinsically stretchable X-ray detectors fabricated using the n-type semiconductor films. **a** The energy level diagrams of CNT electrodes and FIID-CF_3_TVT semiconductor for X-ray detectors. **b** Total mass attenuation of X-rays by FIID-CF_3_TVT hybrid polymer semiconductor with 70 wt% SEBS showing the contribution from photoelectric effect, compton scattering, and pair production. **c** Temporal response of neat FIID-CF_3_TVT films to X-ray upon turning the X-ray source on and off under 3.5 μGy_air_ s^−1^ dose rates. **d** Typical *I*–*V*_DS_ curves of OCSC n-type hybrid semiconductor films under different dose rates at *V*_G_ = 0 V. **e** Device response of OCSC n-type hybrid polymer semiconductor films to X-ray upon turning the X-ray source on and off under different dose rates at *V*_DS_ = 60 V (*V*_G_ = 0 V). **f** X-ray photocurrent density as a function of X-ray dose rate at *V*_DS_ = 60 V (*V*_G_ = 0 V) under serval dose rates from 37.7 to 141 nGy_air_ s^−1^. **g** Performance comparison including the sensitivity (*S*_V_) and detection limit of current reported X-ray detectors—TIPGe-pentacene^46^, TIPS-pentacene^10^, TIPS-pentacene:PS^12^, MEH-PPV^47^, PFO^48^, P3HT^48^ are direct organic X-ray Detectors, B_2_O_3_/P3HT:PCBM^15^, PbS/P3HT:PCBM^16^, Ta/F8T2^49^, B_2_O_3_/PTAA^49^ are direct organic–inorganic hybrid X-ray Detectors, MAPbBr_3-x_Cl_x_^33^, (NH_4_)_3_Bi_2_I_9_^7^, Cs_2_AgBiBr_6_^5^, CsPbBr_3_^4^, MAPbBr_3_^50^, and MAPbI_3_^51^ are perovskite detectors, GOS:Tb/P3HT:PCBM^14^, CsPbBr_3_/P3HT:PCBM^45^ are indirect detectors—with our prepared stretchable X-ray detectors in this work (the single red pentagram, at *V*_DS_ = 10 V). **h** Change in X-ray and dark current density under variable strains as a function of the strain of the measured device at *V*_DS_ = 60 V (*V*_G_ = 0 V). **i** X-ray and dark current density of the device measured after 1, 100, 400, and 1000 stretching-release cycles, respectively, at 25% strain parallel to the charge-transporting direction at *V*_DS_ = 60 V (*V*_G_ = 0 V). The error bars were obtained from six individual devices.

We investigated the X-ray detection capacity of the stretchable X-ray detector with BGTC configuration using n-type polymer semiconductors as the photoactive layer (Fig. 2a). Figure 3a indicates that the well-matched energy levels of the CNT electrodes and FIID-CF_3_TVT polymers were beneficial for the injection and transmission of electrons in the conductive channel. The mass attenuations and absorption coefficients of the neat FIID-CF_3_TVT and hybrid polymer semiconductors were calculated to evaluate the X-ray conversion efficiency through the XCOM database^39^ (Fig. 3b and Supplementary Fig. 19). Moreover, the photoresponse of the neat n-type polymer semiconductor was demonstrated under repeated X-ray switching pulses (Fig. 3c). To decrease the dark current and exclude the influence of mobile charges, the X-ray detection performances of the device were collected at *V*_G_ = 0 V. To the best of our knowledge, photoelectronic effect allows efficient photogeneration of electron-hole pairs, and is subsequently separated by the electric field. The high efficiency of photogenerated carriers and the enhanced dissociation of excitons may collectively contribute to the high photosensitivity of our n-type polymer semiconductor films. Furthermore, the effects of film morphology and carrier mobility on the photoelectronic performances of organic photoelectronic devices have been previously verified^12,40^. The highly aligned hybrid polymer semiconductor films with spatially nanoconfined structures were advantageous towards achieving enhanced stretchability and improved electron-transporting efficiency, and ultimately realized the stretchable high-sensitivity X-ray detector. The drain current (*I*) of the stretchable X-ray detector was linearly dependent on the applied voltage (*V*), demonstrating the ideal Ohmic contact between the hybrid semiconductor film and CNTs electrodes (Fig. 3d). It is observed that the dose rate-dependent photocurrent of the stretchable X-ray detector gradually increased from 3.5 μGy_air_ s^−1^ to 11.97 mGy_air_ s^−1^, which was related to the enhanced concentration of photogenerated charge carriers (Fig. 3d, e). The stretchable hybrid polymer semiconductor exhibited a lower dark current (*I*_dark_ ~1 × 10^−12 ^A, *J*_dark_ ~0.125 nA cm^−2^), a higher *I*_on_/*I*_off_ ratio (~10^2^) and a faster response time (~40 ms) compared with those of the neat polymer (*I*_dark_ ~10^−9 ^A, *J*_dark_ ~125 nA cm^−2^, *I*_on_/*I*_off_ ~3.4, *t*~10 s) (Fig. 3c and Supplementary Fig. 20). Compared with the neat polymer films, the significantly decreased response time of the hybrid polymer films is mainly attributed to the enhanced electron mobility, reduced interface defects and better interfacial contact between the spatially nanoconfined semiconductor layer and the source/drain electrodes. However, both the neat and hybrid semiconductor films showed a long decay time due to the slow detrapping or recombination of photogenerated electron-hole pairs after turning off the X-ray irradiation^12^. Notably, when X-ray radiation was applied, the fast photon absorption of the hybrid polymer semiconductor produced a transient photocurrent that subsequently declined to an equilibrium under electric field redistribution. This overshoot phenomenon was also observed in a previous study on perovskite photodetectors and polymeric solar cells^41^. The lowest detection limit is a significant parameter for evaluating the performance of X-ray detectors. Supplementary Fig. 21 shows that the stretchable X-ray detector could detect the X-ray irradiation as low as 37.7 nGy_air_ s^−1^, which is comparable to the existing record-low value reported for CH_3_NH_3_PbBr_3-x_Cl_x_ perovskite single crystals (7.6 nGy_air_ s^−1^)^33^. Furthermore, we calculated the sensitivity from the slope of the dose-response curve according to six points adjacent to the target dose rate (Fig. 3f and Supplementary Fig. 22). Due to the remarkable non-linear response over a wide range of dose rates, the sensitivities of six different regions could be obtained by fitting the photocurrent densities and dose rates. Surprisingly, a sensitivity value of up to 1.52 × 10^4^ μC Gy_air_^−1^ cm^−2^ (corresponding to the sensitivity value per unit volume of 1.52 × 10^9^ μC Gy_air_^−1^ cm^−3^) was observed for the dose rate of 37.7–141 nGy_air_ s^−1^. However, with increasing irradiation intensity, the sensitivity decreased continuously because of the gradual saturation of current density with smaller gain (Supplementary Fig. 23). The similar phenomenon was also observed in the reported photoconductive and phototransistor-based X-ray detectors^5,10,12,42,43^. Therefore, the ultrahigh sensitivity and photoconductive gain (2.2 × 10^8^) are ascribed to low dark current of the X-ray detectors with stretchable hybrid semiconductor films. To further assess the performance of the stretchable X-ray detectors at low voltage, the devices were also measured at 10 V bias voltage, which could still show good response to X-ray irradiation even at 37.7 nGy_air_ s^−1^ dose rate and exhibited a high sensitivity of 3590 μC Gy_air_^−1^ cm^−2^ (Supplementary Fig. 24). Herein, we also investigated the effects of air ionization and SEBS background current to confirm their contribution to the devices. Indeed, the photocurrent of the neat SEBS is rather lower than the air and hybrid semiconductor (Supplementary Fig. 25), thereby excluding its contribution to the photosensing performances of the prepared STOFET-based X-ray detectors. In addition, the transfer characteristics of STOFETs revealed an ascending current under X-ray irradiation at *V*_DS_ = 60 V (*V*_G_ = 0 V), suggesting that the hybrid semiconductor can efficiently generate charge carriers under X-ray irradiation (Supplementary Fig. 26). We further explored the effects of various SEBS/FIID-CF_3_TVT weight ratios on the photoelectrical characteristics of the STOFETs, and observed that the addition of SEBS components resulted in a drop of dark current without sacrificing the photocurrent (Supplementary Fig. 27). The photosensitivity and detectable X-ray dose rate of the stretchable X-ray detectors in the current study was compared with those reported in literature, as presented in Fig. 3g and Supplementary Fig. 28. The results indicated that the photosensitivity and detection limit of our designed n-type hybrid polymer semiconductor were comparable to that of the conventional rigid perovskite single crystals^33^ (Supplementary Tables 3 and 4).

For wearable electronic devices, their stabilities (including long-term stability, environmental stability, mechanical stability, etc.) are of crucial significance for practical applications. The stretchable X-ray detector exhibited relatively stable photoelectronic responses when subjected to 30 continuous X-ray switching pulses as shown in Supplementary Fig. 29. In addition, the photocurrent still remained constant even after storage in ambient air for one week, demonstrating its good environmental stability (Supplementary Fig. 30). Figure 3h shows the photoelectronic stability of the stretchable X-ray detector under different strains. When the 0~100% stretching strain was applied along or perpendicular to the electron-transporting direction, the device exhibited a gradually increased photocurrent which is because the increased channel areas under stretching strains contributed to the charge collection efficiency (Fig. 3h and Supplementary Fig. 31). To confirm this, we analyzed the photocurrent changes under different channel film thicknesses which exhibited no apparent change due to the nanoscale thinning of the active films (Supplementary Fig. 32). In addition, the strain-induced crystallization^44^ and aligned mechanisms^45^ can be also the major contribution to the improved photocurrent. To further describe the cycling stability of the stretchable X-ray detector, the device was subjected to 1000 continuous stretching-releasing cycles at 25% strain along the electron-transporting direction, and it still sustained a relatively stable photocurrent and *I*_on_/*I*_off_ (Fig. 3i and Supplementary Fig. 33). The slightly increased photocurrent can be attributed to the improved crystallinity, enabled by the chain re-arrangement under strain and partial ‘permanent deformation’ in hybrid polymer films after multiple stretching-releasing cycles^44^ (Supplementary Fig. 34). On the basis of all the analyses above, it could be concluded that the prepared stretchable X-ray detector has an excellent photoelectrical performance, superior strain tolerance, and long-term stability, which makes it suitable for skin-like photoelectronic devices.

### The X-ray imaging of polymer films

**Fig. 4:**
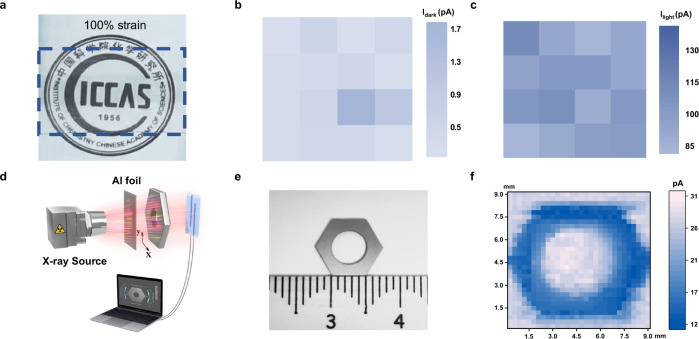
X-ray imaging of the intrinsically stretchable n-type semiconductor films. **a** Images showing high transparency and stretchability of the hybrid semiconductor film. **b** The dark current and **c** the X-ray photocurrent extracted from 16 working devices, respectively. All the devices biased at *V*_DS_ = 60 V (*V*_G_ = 0 V), were irradiated with a dose rate of 3.5 μGy_air_ s^−1^. **d** Schematic representation of the single-pixel scanning imaging devices (Al foil with 4.8 mm thickness worked as the attenuator). **e** Photograph of a hexagonal nut with sides of 5 mm length. **f** Experimental results of the imaged object under X-rays of 25 keV with a dose rate of 3.65 μGy_air_ s^−1^. The imaging device were biased at *V*_DS_ = 10 V (*V*_G_ = 0 V).

To demonstrate the imaging capacity of the hybrid semiconductor films (Fig. 4a), it is of great significance to investigate the device performance uniformity. No obvious discrepancy was observed in the dark current and photocurrent of 16 devices, as shown in Fig. 4b, c. Considering the lack of scalable fabrication technology and platform for stretchable X-ray detectors, we here performed the single-pixel X-ray imaging characterization of the hybrid polymer semiconductor films. Figure 4d shows the single-pixel imaging devices with a computer-controlled translation system (X–Y direction) of the objects. Even at a low-dose rate (3.65 μGy_air_ s^−1^, which is a record-low value among the reported organic X-ray detector^10^), the prepared hybrid polymer semiconductor successfully achieved good X-ray imaging of a hexagonal nut (Fig. 4e, f). In addition, the X-ray imaging based on stretchable polymer semiconductors was achieved at approximately one-twelfth dose rate of commercial medical chest X-ray diagnosis^19^. Consequently, the development of stretchable X-ray detectors prepared using n-type hybrid polymer semiconductors with good mechanical ductility, skin conformability, and photoelectronic performances offers great prospects for low-dose imaging of organisms.

## Discussion

To conclude, we demonstrated a controlling method for intrinsically stretchable n-type hybrid polymer semiconductors and achieved a skin-like X-ray detector. The designed spatially nanoconfined morphology and highly aligned polymer nanofiber networks collectively ensure high electron-transporting efficiency, stability, and strain-tolerance capacity. As a result, the optimized stretchable hybrid polymer semiconductor exhibited an ultrahigh photosensitivity of 1.52 × 10^4^ μC Gy_air_^−1^ cm^−2^, an ultralow detection limit of 37.7 nGy_air_ s^−1^, and excellent mechanical stability of 1000 stretching cycles at 25% strain. Compared with the control experiment, the fabricated stretchable n-type hybrid polymer semiconductor film simultaneously showed a fast response time of 40 ms and a high *I*_on_/*I*_off_ of 10^2^, which benefited from the enhanced electron transport, especially the significantly reduced subthreshold swing. Significantly, the polymer film X-ray imaging was documented at a record-low-dose rate of 3.65 μGy_air_ s^−1^, signifying its enormous potential for use in skin-like X-ray detection and imaging in wearable and implantable monitors.

## Methods

### Materials

The styrene-ethylene butylene styrene (SEBS-H1221 and SEBS-H1052 with volume fractions of 88% and 80% poly(ethylene-co-butylene), respectively) was purchased from Asahi Kasei (Japan). SEBS-H1221 was employed as the substrate and elastic component in the stretchable hybrid polymer semiconductors, and SEBS-H1052 was only used to fabricate the dielectric layer. Single-walled carbon nanotubes (P3-SWNTs) were purchased from Carbon Solutions for the electrode (including gate, drain, and source electrodes) fabrication. The conjugated polymers (FIID-CF_3_TVT) with the number-averaged molecular weight of 25.5 kDa and polydispersity of 1.9 Ð were synthesized from our laboratory. Poly(dimethylsiloxane) (PDMS, Sylgard 184) was purchased from Dow Corning. PDMS substrates were fabricated at the weight ratio of 10:1 and 20:1 (base:cross-linker, w-w), and cured at 100 °C for 1 h (Supplementary Fig. 5). All solvents, including xylene, toluene, chlorobenzene, and cyclohexane were purchased from J&K Seal and were used as received.

### Film and device fabrication

The OTS-modified Si wafers were prepared to deposit each functional layer. Firsty, Si wafers were soaked in the piranha solution (H_2_SO_4_: H_2_O_2_ = 7:3) for 20 min, and then ultrasonicated in deionized water, acetone, and ethanol for 7 min. After that, the Si/SiO_2_ wafers were dried with N_2_ and treated in O_2_ plasma for 5 min, and then modified using OTS molecules in vacuum oven at 120 °C for 3 h.

#### Preparation of semiconducting films

The polymer semiconductor films were prepared by depositing the 10 mg mL^−1^ FIID-CF_3_TVT: SEBS-H1221 solution in xylene on the OTS-modified Si/SiO_2_ wafers based on the OCSC method (Fig. 1a), where the substrate (7 × 12 mm^2^) was placed with its center away from the rotation axis of the spin-coater at a distance of 20–40 mm. During the OCSC process, the spinning speed gradually increased to 2000 rpm, followed by annealing at 220 °C for 10 min.

#### Preparation of electrodes

The CNT electrodes including gate, source and drain electrodes are prepared by spray-coating the 0.25 mg mL^−1^ of P3-SWNTs/isopropanol solution on the OTS-modified Si/ SiO_2_ wafers attached with a patterned mask tightly. During the spray-coating, the wafer was placed on a hot stage at 120 °C with a distance between the spray gun and the wafer of 8–15 cm.

#### Preparation of dielectric

The dielectric layers were prepared by depositing the 65 mg mL^−1^ SEBS/cyclohexane solution on the OTS-modified Si/ SiO_2_ wafers with a spinning speed of 1000 rpm and an annealing temperature of 80 °C for 1 h. The resultant dielectric layer has a thickness of about 2000 nm and an approximate capacitance of 0.94 nF cm^−2^ (Supplementary Table. 3).

#### Preparation of substrates

The substrate and encapsulation layer were prepared by dropping SEBS/toluene solution of 200 mg mL^−1^ and 120 mg mL^−1^ onto the OTS-modified glass sheets, respectively, followed by annealing at 40 °C for 5 h. The thickness of substrate and encapsulation layer were ~800 μm and 200 μm, respectively.

#### Preparation of devices

The rigid field-effect transistors with a BGTC stack were fabricated by preparing the polymer semiconductor films on OTS-modified Si/ SiO_2_ wafer. Then, the source and drain electrodes (channel width/length = 4000/200 µm) were evaporated with an Au film thickness of 30 nm on the top of polymer semiconductors. The STOFETs for both electrical characteristics and X-ray detection with BGTC configuration were fabricated based on the sequential thermal lamination-transfer process, which was beneficial for reducing the interlayer bubbles and defects. The patterned gate electrode, SEBS dielectric, polymer semiconductor, and patterned source/drain electrodes (channel width/length = 4000/200 µm) were sequentially transferred by the SEBS substrate from OTS-modified Si/SiO_2_ wafers (Supplementary Fig. 10). The lamination processes were in a vacuum oven at 50 °C for 30 min. All the lamination-transfer processes were conducted in ambient atmosphere.

### Characterization

UV–vis absorption measurements were conducted using a SHIMADZU 2600 UV–vis spectrophotometer. The polarized-absorption spectrum was measured by the spectrophotometer combined with a polarizer. GIXRD was performed at shanghai synchrotron radiation facility (SSRF) on beamline NO. 14 B/15 U. GIWAXS images were collected by a Xenocs Xeuss 2.0 with Cu Kα radiation. AFM measurement was conducted by a Bruker atomic force microscopy. Optical microscope images were obtained from a BX53 cross-polarized optical microscope. The water and diiodomethane contact angle were measured by Drop Shape Analyzer (DSA100, KRÜSS) in static mode at room temperature. The obtained contact angle calculated by averaging the left and right angles of a sessile drop using the KRÜSS software based on the tangential method. All the films were characterized by an ambient atmosphere.

### Optoelectrical measurements

The electrical characteristics were characterized by the Keithley 4200 in vacuum. X-ray detection experiments were performed by the PDA FS380 in ambient conditions using Moxtek MADPRO (60 kV 12 W) as the X-ray source. The X-ray beam generated the focal spot with a height of 1.26 mm and a width of 0.30 mm. The height between the detector and the X-ray source was 10 cm, and the aluminum foil with a thickness of 4.8 mm that worked as the attenuator was inserted between the X-ray source and the device. The attenuated dose rates for different current and voltage of X-Ray tube were calibrated by the X-ray dosimeter (Model: MagicMax, IBA, German).

### Capacitance measurement

The capacitance measurements were performed with parallel plate capacitor structures on a SEBS substrate. CNTs and SEBS-H1052 was used as the electrodes and dielectric layer (~2000 nm), respectively. The facing area of the capacitors between the upper electrode and bottom electrode was determined to be 0.008 cm^2^ (length/width = 4000/200 µm) that is consistent with the channel area of STOFETs. The capacitance data as a function of frequency were acquired by a Keysight B2900 Precision LCR Meter. The capacitances under different strains are all averaged values under 1000 Hz from at least five devices.

### Single-pixel scanning imaging system

The single-pixel imaging experiments were carried out on the optical platform connecting with a stepping-motor controlled translation (X–Y) stage in air. A stainless steel sheet of the hexagonal nut with an edge length, inner radius, and thickness of 5 mm as the imaging object was fixed on the x–y translation system. The distance between the imaging object and the device was 0.5 mm, and the channel length/width was 5/1400 µm. The response current of the device (*W* = 1400 μm and *L* = 5 μm) was recorded using the Keysight B2900A according to the coordinates that were determined by moving the object every 300 µm along the *x* axis and 300 µm along the y-axis, respectively.

## Supplementary information

Supplementary Information

## Data Availability

The authors declare that all data supporting the findings of this study are available within the paper and its Supplementary Information. Source data are provided with this paper.

## Source data

Source Data

## References

[1] Dai Y (2021). Stretchable transistors and functional circuits for human-integrated electronics. Nat. Electron..

[2] Wang W (2021). Strain-insensitive intrinsically stretchable transistors and circuits. Nat. Electron..

[3] Kim YC (2017). Printable organometallic perovskite enables large-area, low-dose X-ray imaging. Nature.

[4] Pan W (2019). Hot-pressed CsPbBr_3_ quasi-monocrystalline film for sensitive direct X-ray detection. Adv. Mater..

[5] Pan W (2017). Cs_2_AgBiBr_6_ single-crystal X-ray detectors with a low detection limit. Nat. Photon..

[6] Zhao J (2020). Perovskite-filled membranes for flexible and large-area direct-conversion X-ray detector arrays. Nat. Photon..

[7] Zhuang R (2019). Highly sensitive X-ray detector made of layered perovskite-like (NH_4_)_3_Bi_2_I_9_ single crystal with anisotropic response. Nat. Photon..

[8] Liang C (2020). Thermoplastic membranes incorporating semiconductive metal–organic frameworks: an advance on flexible X-ray detectors. Angew. Chem. Int. Ed..

[9] Cheng L (2020). Three-dimensional polycatenation of a uranium-based metal–organic cage: structural complexity and radiation detection. J. Am. Chem. Soc..

[10] Basiricò L (2016). Direct X-ray photoconversion in flexible organic thin film devices operated below 1V. Nat. Commun..

[11] Ciavatti A (2015). Toward low-voltage and bendable X-ray direct detectors based on organic semiconducting single crystals. Adv. Mater..

[12] Temiño I (2020). Morphology and mobility as tools to control and unprecedentedly enhance X-ray sensitivity in organic thin-films. Nat. Commun..

[13] Chen M (2021). Organic semiconductor single crystals for X-ray imaging. Adv. Mater..

[14] Büchele P (2015). X-ray imaging with scintillator-sensitized hybrid organic photodetectors. Nat. Photon..

[15] Thirimanne HM (2018). High sensitivity organic inorganic hybrid X-ray detectors with direct transduction and broadband response. Nat. Commun..

[16] Ankah GN (2016). PbS quantum dot based hybrid-organic photodetectors for X-ray sensing. Org. Electron..

[17] Nanayakkara MPA (2021). Ultra-low dark current organic–inorganic hybrid X-ray detectors. Adv. Funct. Mater..

[18] Gao Y (2021). Ultrathin and ultrasensitive direct X-ray detector based on heterojunction phototransistors. Adv. Mater..

[19] Liu K (2021). Ultrahigh-performance optoelectronic skin based on intrinsically stretchable perovskite-polymer heterojunction transistors. Adv. Mater..

[20] Nikzad S (2020). Inducing molecular aggregation of polymer semiconductors in a secondary insulating polymer matrix to enhance charge transport. Chem. Mater..

[21] Oh JY (2016). Intrinsically stretchable and healable semiconducting polymer for organic transistors. Nature.

[22] Zheng Y (2019). An intrinsically stretchable high-performance polymer semiconductor with low crystallinity. Adv. Funct. Mater..

[23] Xu J (2017). Highly stretchable polymer semiconductor films through the nanoconfinement effect. Science.

[24] Zhang G (2017). Versatile interpenetrating polymer network approach to robust stretchable electronic devices. Chem. Mater..

[25] Wang SH (2018). Skin electronics from scalable fabrication of an intrinsically stretchable transistor array. Nature.

[26] Choi D (2016). Elastomer–polymer semiconductor blends for high-performance stretchable charge transport networks. Chem. Mater..

[27] Park Y (2021). Skin-like low-noise elastomeric organic photodiodes. Sci. Adv..

[28] Sim K (2019). Fully rubbery integrated electronics from high effective mobility intrinsically stretchable semiconductors. Sci. Adv..

[29] Guan Y-S (2020). Air/water interfacial assembled rubbery semiconducting nanofilm for fully rubbery integrated electronics. Sci. Adv..

[30] Sun H, Guo X, Facchetti A (2020). High-performance n-type polymer semiconductors: applications, recent development, and challenges. Chem.

[31] Ran Y (2021). Nonchlorinated solubility enhanced by lipophilicity: an effective strategy for environmentally benign processing of rigidly regular n-type polymeric semiconductors. Adv. Electron. Mater..

[32] Sodeoka M (2011). Efficient fluorination of organic molecules with chiral anions. Science.

[33] Wei H (2017). Dopant compensation in alloyed CH_3_NH_3_PbBr_3−x_Cl_x_ perovskite single crystals for gamma-ray spectroscopy. Nat. Mater..

[34] Ko J (2021). Electronic effects of nano-confinement in functional organic and inorganic materials for optoelectronics. Chem. Soc. Rev..

[35] Xu J (2019). Multi-scale ordering in highly stretchable polymer semiconducting films. Nat. Mater..

[36] Yuan Y (2014). Ultra-high mobility transparent organic thin film transistors grown by an off-centre spin-coating method. Nat. Commun..

[37] Yang, J. et al. A nonchlorinated solvent-processed polymer semiconductor for high-performance ambipolar transistors. *Natl. Sci. Rev*. **9**, nwab145 (2021). 10.1093/nsr/nwab145 PMC9031015 35475218

[38] Jiang Y (2021). Alignment of linear polymeric grains for highly stable N-type thin-film transistors. Chem..

[39] Berger, M. J. et al. XCOM: Photon Cross Sections Database: NIST Standard Reference Database 8 (NIST, 2013). https://www.nist.gov/pml/xcom-photoncross-sections-database.

[40] Guo YL, Yu G, Liu YQ (2010). Functional organic field-effect transistors. Adv. Mater..

[41] Chen S (2016). A flexible UV–vis–NIR photodetector based on a perovskite/conjugated-polymer composite. Adv. Mater..

[42] Liu J (2019). Flexible, printable soft-x-ray detectors based on all-inorganic perovskite quantum dots. Adv. Mater..

[43] Pan L, Shrestha S, Taylor N, Nie W, Cao LR (2021). Determination of X-ray detection limit and applications in perovskite X-ray detectors. Nat. Commun..

[44] Xu, J. et al. Tuning conjugated polymer chain packing for stretchable semiconductors. *Adv. Mater*. **34**, 2104747 (2021). 10.1002/adma.202104747 34558121

[45] Xiang L (2021). X-ray Sensitive hybrid organic photodetectors with embedded CsPbBr3 perovskite quantum dots. Org. Electron..

[46] Ciavatti A (2018). Boosting direct X-ray detection in organic thin films by small molecules tailoring. Adv. Funct. Mater..

[47] Boroumand FA (2007). Direct x-ray detection with conjugated polymer devices. Appl. Phys. Lett..

[48] Valitova I (2021). Poly(3-hexylthiophene−2,5-diyl) based diodes for ionizing radiation dosimetry applications. Org. Electron..

[49] Mills CA (2013). Enhanced x-ray detection sensitivity in semiconducting polymer diodes containing metallic nanoparticles. J. Phys. D: Appl. Phys..

[50] Wei W (2017). Monolithic integration of hybrid perovskite single crystals with heterogenous substrate for highly sensitive X-ray imaging. Nat. Photon..

[51] Yakunin S (2016). Detection of gamma photons using solution-grown single crystals of hybrid lead halide perovskites. Nat. Photon..

