# Supplementary material for: Spatially nanoconfined N-type polymer semiconductors for stretchable ultrasensitive X-ray detection
Source: Nat Commun. 2022 Nov 22;13:7163. doi: 10.1038/s41467-022-34968-1 (PMC9684452; doi:10.1038/s41467-022-34968-1)
Supplement: Supplementary file 1 — Supplementary Information [file 41467_2022_34968_MOESM1_ESM.pdf]

SUPPLEMENTARY INFORMATION

Spatially Nanoconfined N-type Polymer Semiconductors for Stretchable Ultrasensitive X-Ray Detection

Yangshuang Bian, Kai Liu, Yang Ran, Yi Li, Yuanhong Gao, Zhiyuan Zhao, Mingchao Shao, Yanwei Liu, Junhua Kuang, Zhiheng Zhu, Mingcong Qin, Zhichao Pan, Mingliang Zhu, Chengyu Wang, Hu Chen, Jia Li, Xifeng Li, Yunqi Liu and Yunlong Guo*

*Corresponding author. Email: guoyunlong@iccas.ac.cn (Y.G.)

Section 1. Surface energies of the conjugated polymer and SEBS elastomer used in this study:

The water and diiodomethane contact angle of polymer semiconductor films were measured by the Drop Shape Analyzer (DSA100, KRÜSS) in static mode at room temperature. The obtained contact angle from the average of the left and right angles of a sessile drop was measured by KRÜSS software based on the tangential method. The de-ionized water and diiodomethane (≈ 1 μL) were dropped on the neat and hybrid semiconductor film, and then the droplet was snapshotted after the equilibrium on the gas-liquid-solid interface. The contact angle need to be fixed at the standard deviation of ±0.5°.[1, 2] The surface energy of polymer semiconductor films was calculated by the Owens-Wendt method equation[3]:

$$\gamma_s = \gamma_s^p + \gamma_s^d$$

$$(1 + cos\theta_l)\gamma_l = 2(\sqrt{\gamma_l^d \gamma_s^d} + \sqrt{\gamma_l^p \gamma_s^p})$$

Where θ_l represents the contact angle of the testing liquid, including water and diiodomethane. Thereinto,γ_s, γ_s^p and γ_s^d are the total surface energy, polar component and dispersion component of the sample, respectively. The total surface energy (γ_l), dispersion component (γ_l^d)and polar component (γ_l^p) are 50.8 mJ m^{-2}, 50.42 mJ m^{-2} and 0.38 mJ m^{-2} for diiodomethane, and 72.8 mJ m^{-2}, 29.1 mJ m^{-2} and 43.7 mJ m^{-2} for water, respectively.

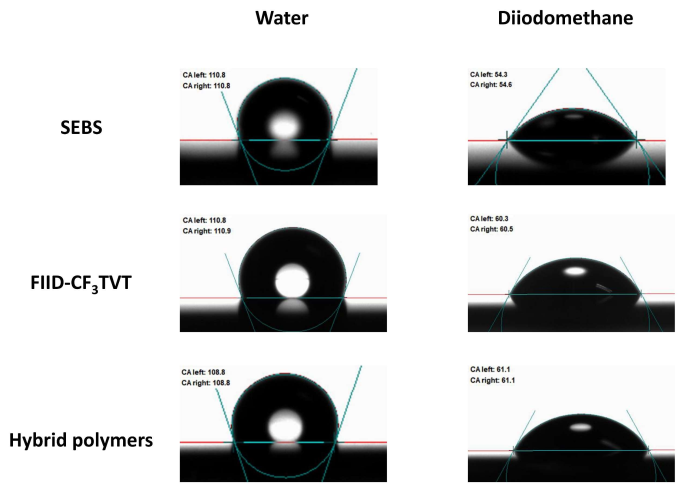

Supplementary Figure 1. Optical images of the snapshotted droplet showing the contact angels of the SEBS, FIID-CF$_3$TVT and nanoconfined hybrid polymer (70 wt% SEBS) films.

Supplementary Table 1. Surface energies of the SEBS, neat FIID-CF$_3$TVT and nanoconfined hybrid polymer (70 wt% SEBS) films.

**Materials**	**Contact angle (˚)**		**Surface energy (mJ/m^2)**		
	CH_2I_2	H_2O	γ_s^d	γ_s^p	γ_s
SEBS	53.5	110.8	32.5	1.21	33.71
FIID- CF$_3$TVT	60.4	110.8	28.9	0.3	29.2
**Hybrid polymers**	**61. 1**	**108.8**	**27.7**	**0.26**	**28.96**

Section 2. The spatial nanoconfinement effect on n-type hybrid polymer films:

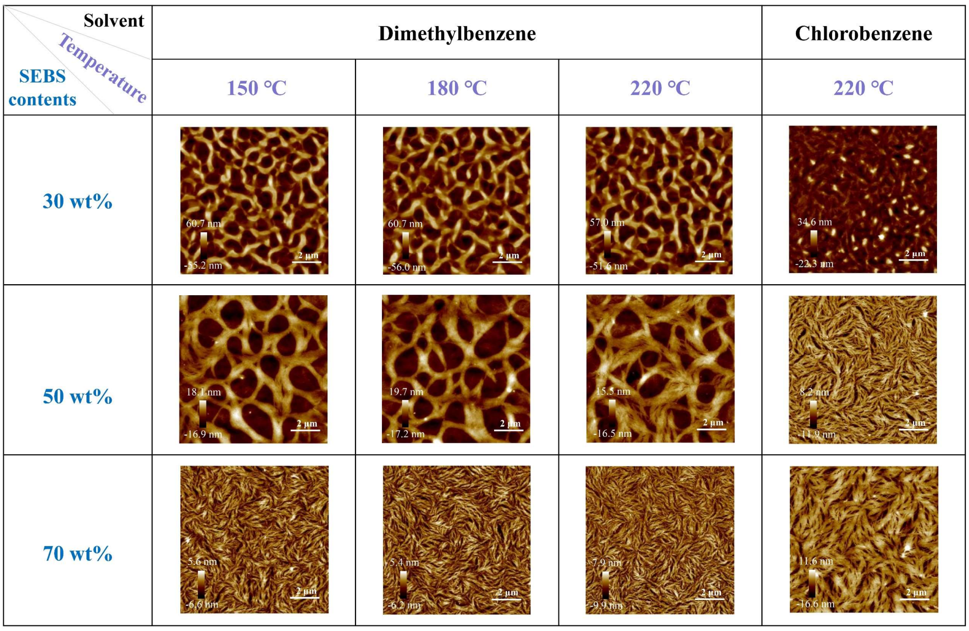

Supplementary Figure 2. Morphology of the hybrid polymer films with different SEBS/FIID-CF_3TVT ratios, annealing temperatures and solvent types.

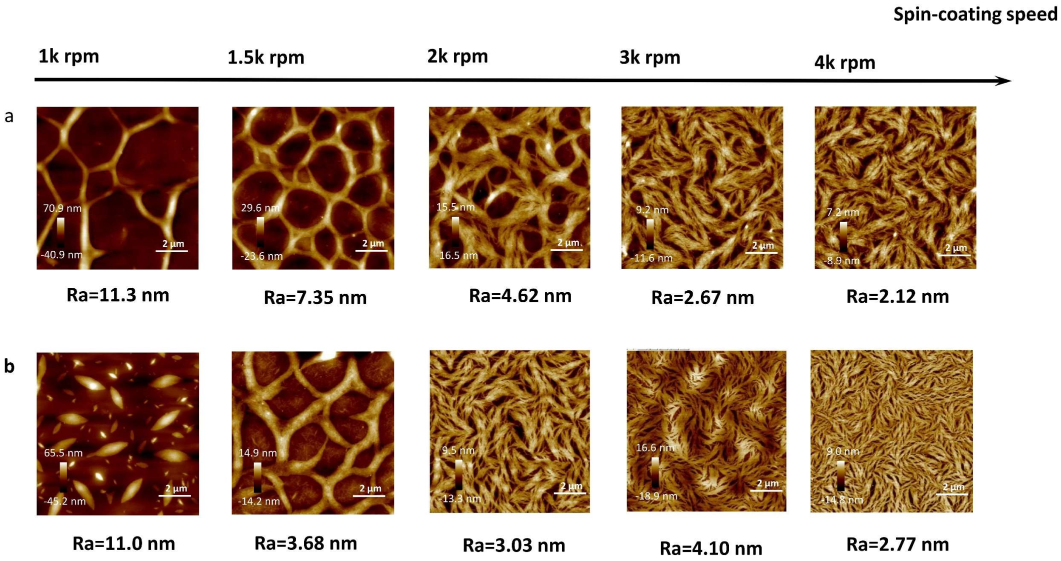

Supplementary Figure 3. Morphology of the hybrid polymer films with the SEBS contents of 50 wt% (a) and 70 wt% (b) at different spin-coating speeds.

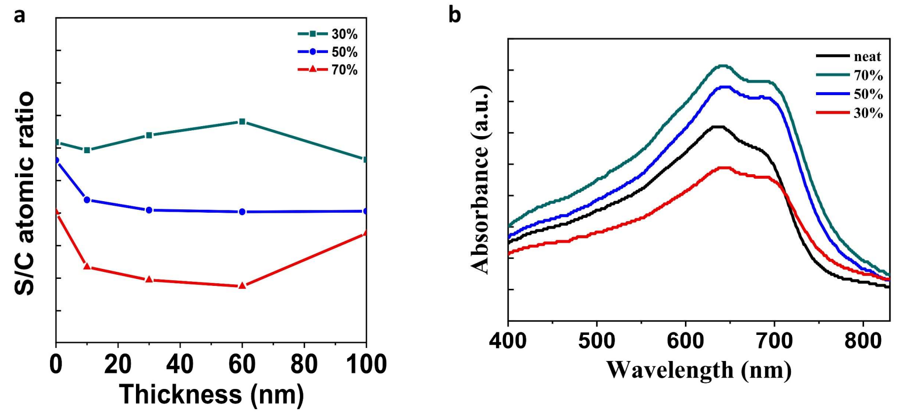

Supplementary Figure 4. Characterization of the neat film and hybrid polymer films with different SEBS contents. (a) Ratios between S_{2p} peak and C_{1s} peak (S/C ratio) from XPS spectra at different depths of three hybrid films. (b) UV-vis spectrum of the neat and hybrid films.

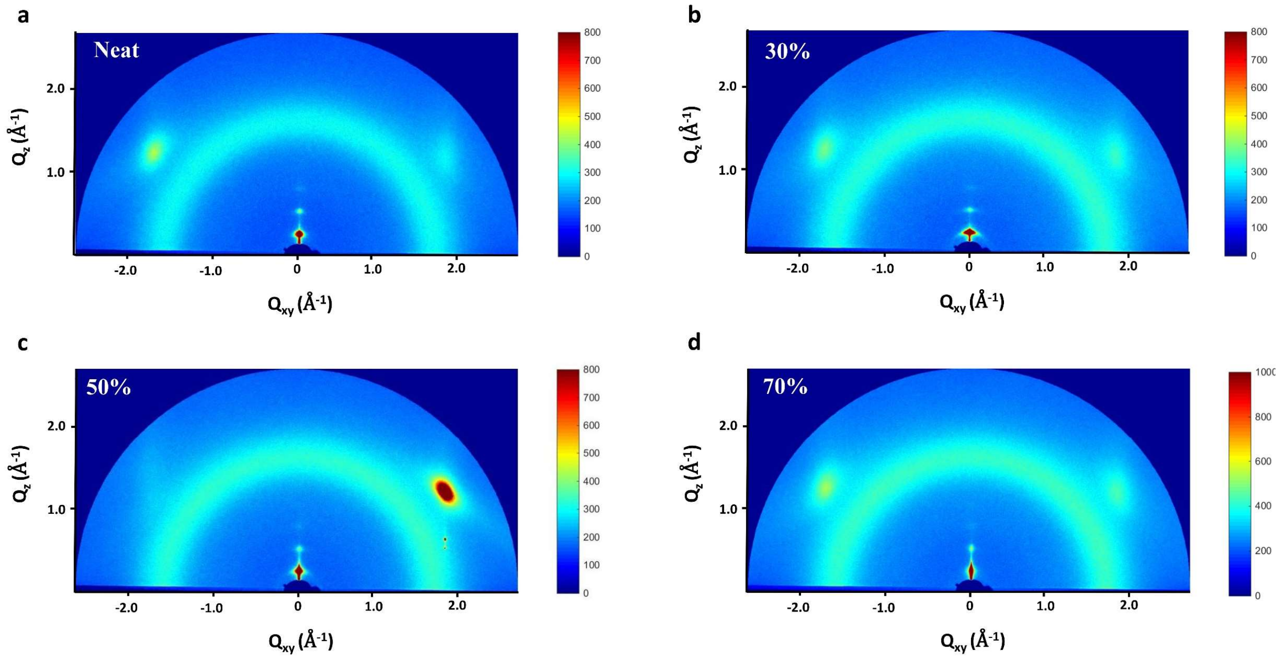

Supplementary Figure 5. Two-dimensional GIXRD pattern of the neat FIID-CF_3TVT (a) and the hybrid polymer films with different SEBS contents of 30 wt% (b), 50 wt% (c) and 70 wt% (d).

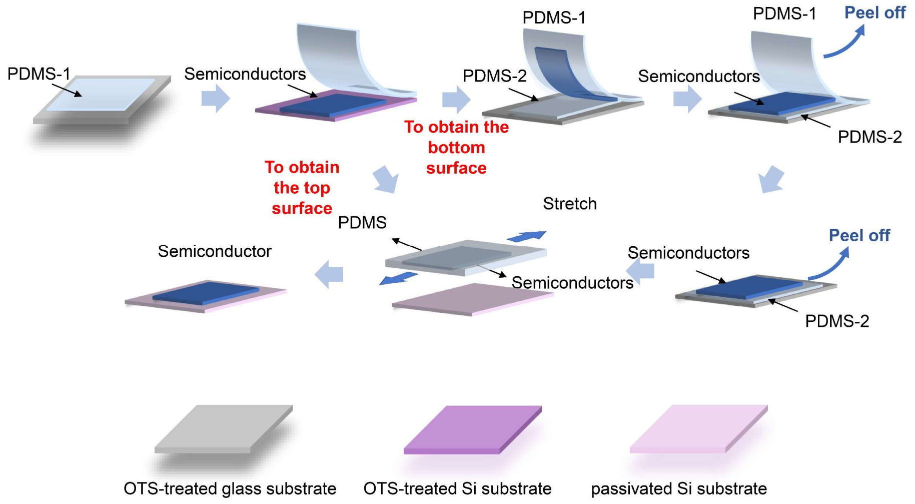

Supplementary Figure 6. Experimental procedure of the soft contact lamination method to obtain both the top and bottom surface morphology of polymer films and the stretched films.

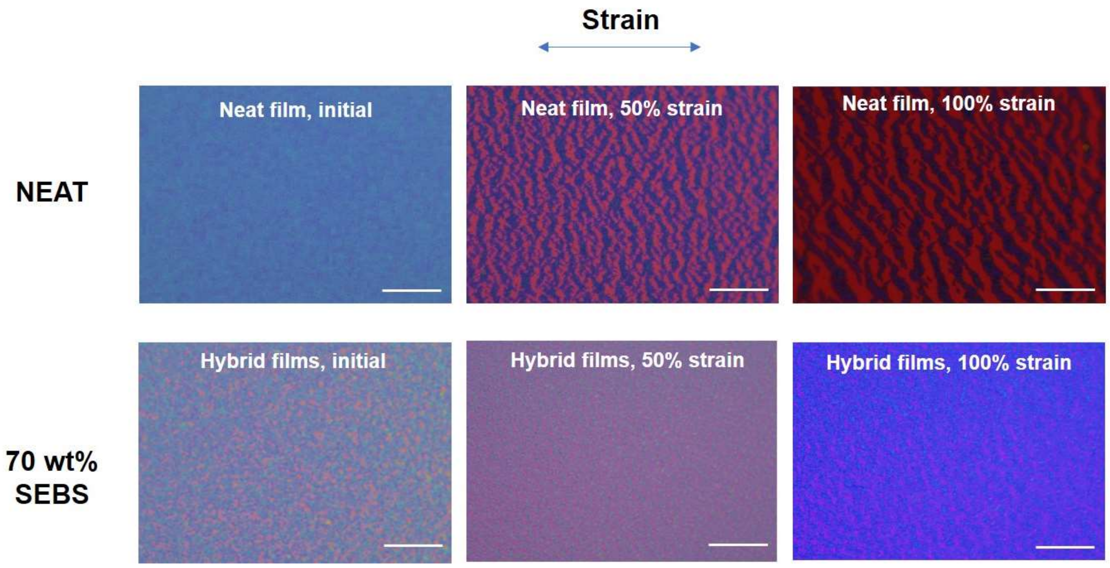

Supplementary Figure 7. Optical microscope images of the neat film and the hybrid polymer film with 70 wt% SEBS under 0% , 50% and 100% stretching strain. The scale bar is 20 µm.

Section 3. The electronic performance of the OFETs fabricated on OTS-modified 300-nm SiO_2/Si wafer and the fully stretchable TFTs:

The mobility (μ), threshold voltage (V_{th}) and subthreshold swing (SS) are important parameters for evaluating the electronic performances of OFETs. These values can be extracted from the curve of the drain current (I_D) versus the gate voltage (V_G). In the saturation regime, the drain current (I_D) versus the gate voltage (V_G) according to the following equation[4]:

$$I_{DS} = \frac{W}{2L} C_i \mu_{sat} (V_G - V_{th})^2$$

Where W and L represent the channel length and width, respectively. C_i is the capacitance of the gate dielectric.

In details, the mobility is calculated by the slop of $\sqrt{|I_{DS}|}$ versus V_G according to the following equation[5]:

$$\mu_{sat} = \left(\frac{\partial\sqrt{|I_{DS}|}}{\partial V_G}\right)^2 \frac{2L}{WCi}, \text{ applicable at } |V_G - V_{th}| < |V_{DS}|$$

The V_{th} of the device is obtained by extrapolating a plot of $\sqrt{|I_{DS}|}$ versus V_G to $I_{DS} = 0$.

The subthreshold swing is defined as:

$$SS = d V_G / d (\log I_{DS})$$

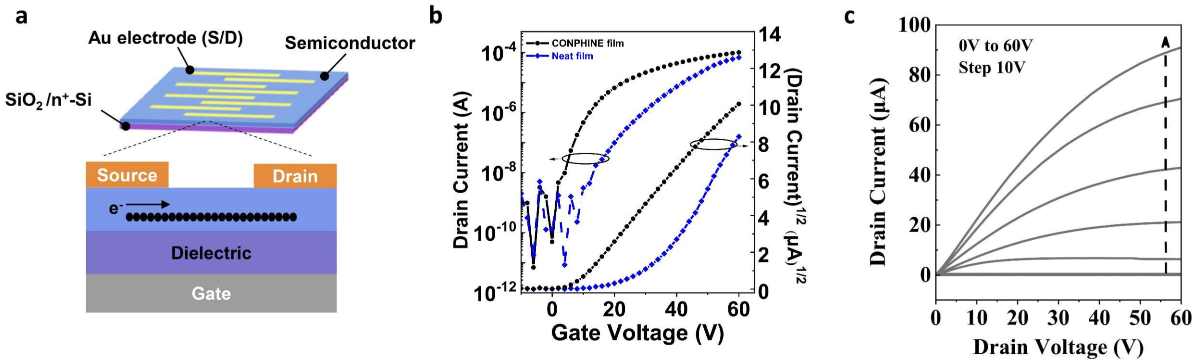

Supplementary Figure 8. The electronic performance of the hybrid polymer films fabricated on OTS-modified 300-nm SiO_2/Si wafer. (a) Schematic diagram of the OFET with a BGTC configuration. (b) Transfer curves (V_{DS} = 60 V) obtained from the neat film and the hybrid polymer film. (c) Output curves of the hybrid polymer film.

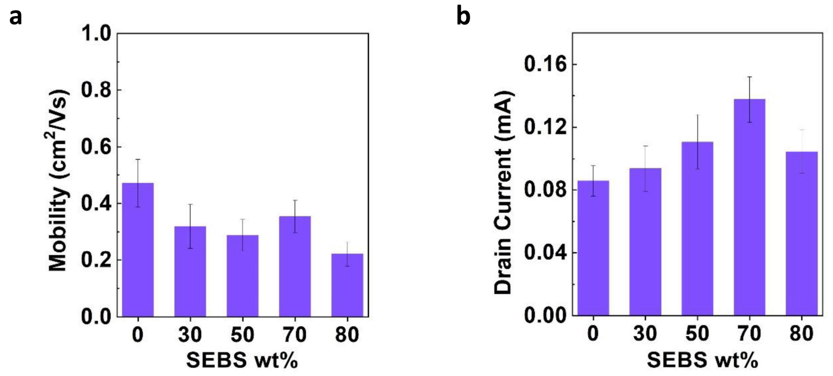

Supplementary Figure 9. Influence of the SEBS fraction on the mobility (a) and drain current (b) of the hybrid polymer films.

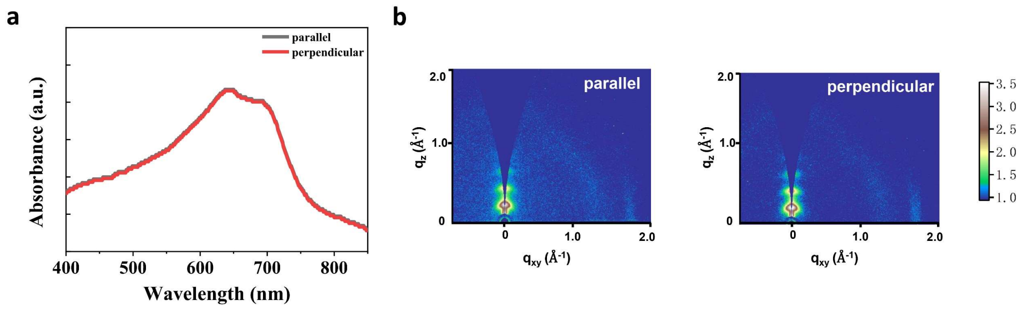

Supplementary Figure 10. Characterization of the hybrid polymer films prepared by the spin-coating (SC) method. (a) The polarized ultraviolet–visible absorption spectroscopy of the SC hybrid polymeric semiconductor film parallel and perpendicular to the radial coating direction. (b) GIWAXS images of the SC hybrid polymer film with the incident beam oriented parallel (left) and perpendicular (right) to the radial coating direction.

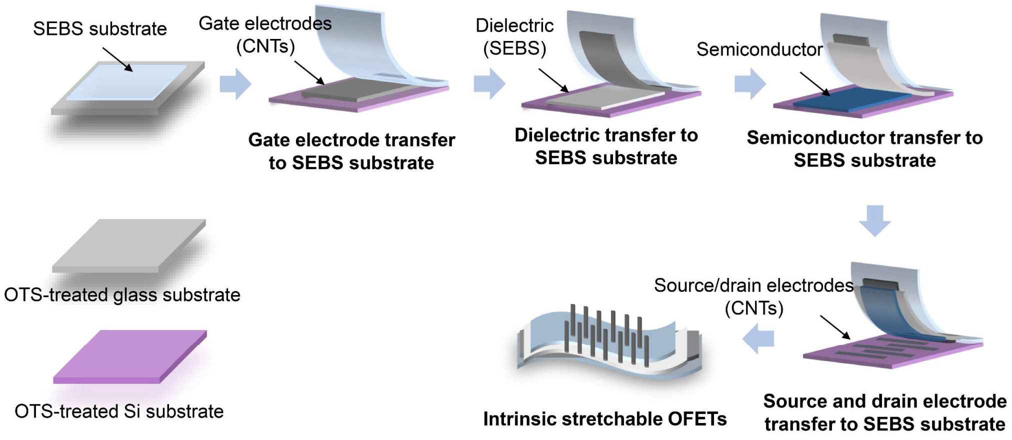

Supplementary Figure 11. Diagram illustrating the fabrication process of fully stretchable devices. The thickness of SEBS substrate can be altered from ~ 10 μm (spin-coating method) to ~ 1 mm (drop-casting method) depending on the practical requirements. The thickness of the electrode is 30~40 nm. The thickness of the dielectric layer and semiconductor layer are 2000 nm and 100 nm, respectively.

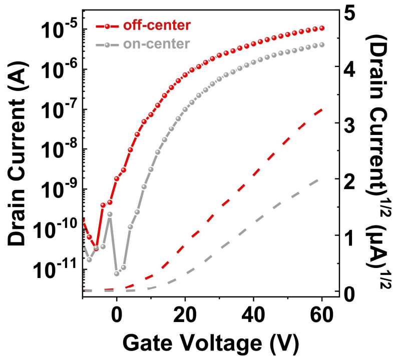

Supplementary Figure 12. The electrical characterization of the fully stretchable transistor using the hybrid polymer semiconductor films prepared by the SC and OCSC methods.

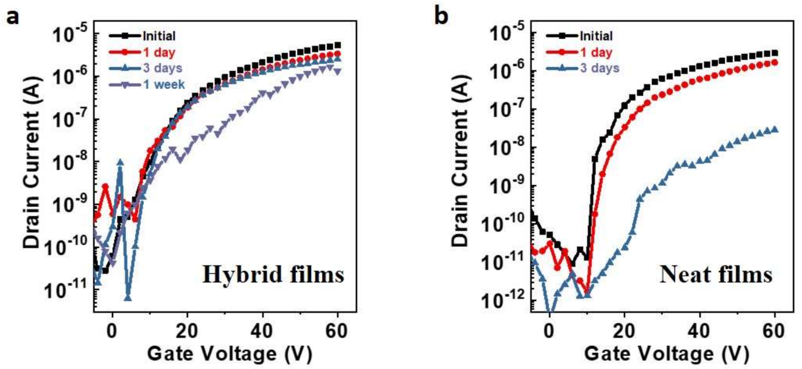

Supplementary Figure 13. The real 'working lifetime' of the hybrid (a) and neat (b) semiconductor films when stored for 1day, 3 days and 1week.

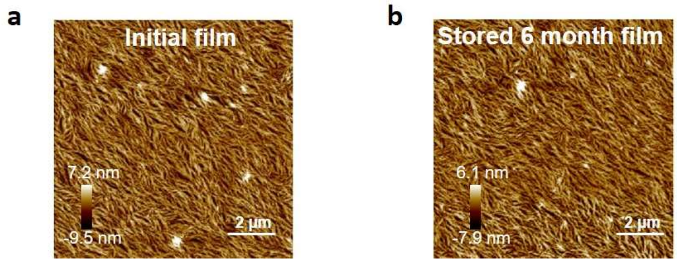

Supplementary Figure 14. AFM phase images of the bottom surface morphology of the hybrid film prepared by the OCSC methods when stored for ~6 months.

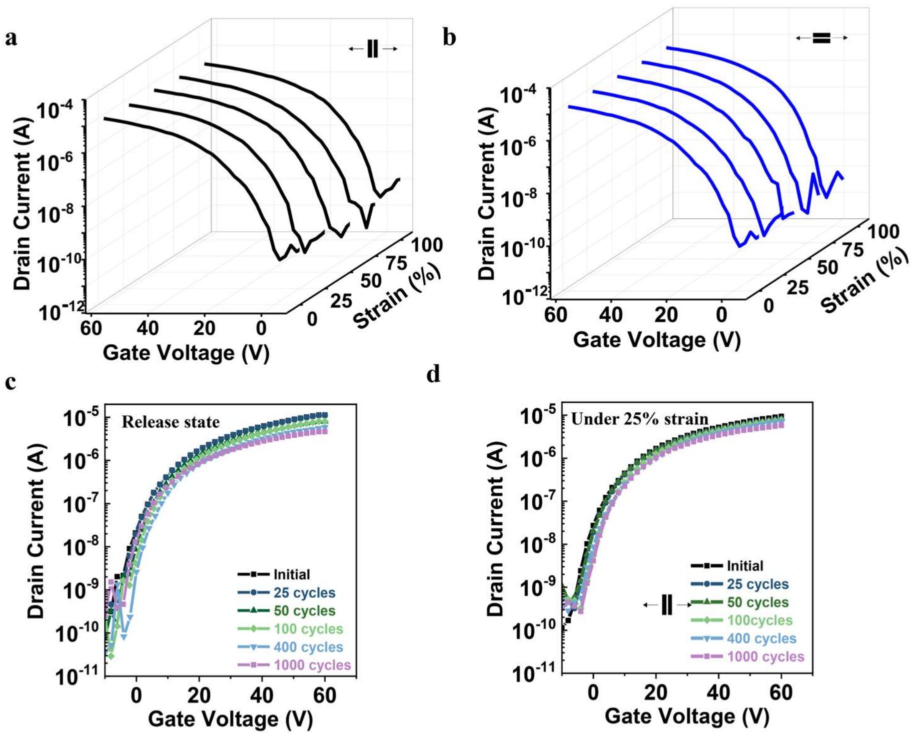

Supplementary Figure 15. Transfer curves (V_{DS} = 60 V) of the STOFETs using OCSC hybrid polymer semiconductor films under different stretching strains and multiple stretch-release cycles. Transfer curves of the STOFETs at different stretching strains (a) parallel and (b) perpendicular to the charge transport direction. Transfer curves of the STOFETs under multiple stretching cycles at 25% strain, parallel to the charge transport direction at the released state (c) and at 25% strain (d).

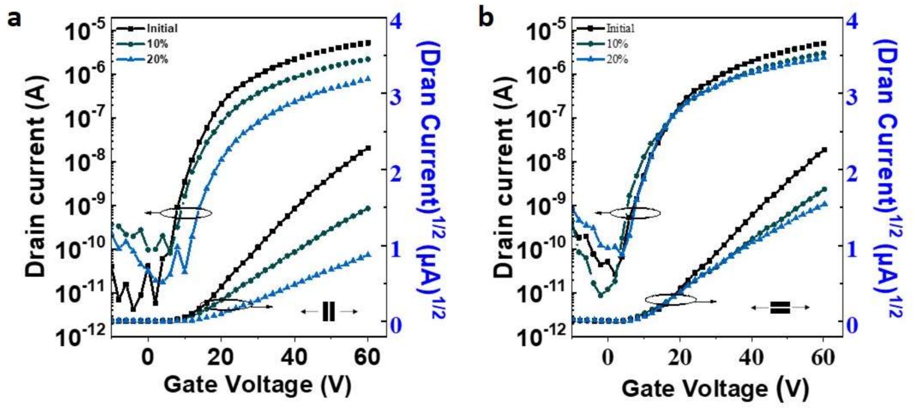

Supplementary Figure 16. Transfer curves (V_{DS} = 60 V) of the neat semiconductor films under different stretching strains along the charge transport direction (a) and perpendicular to the charge transport direction (b).

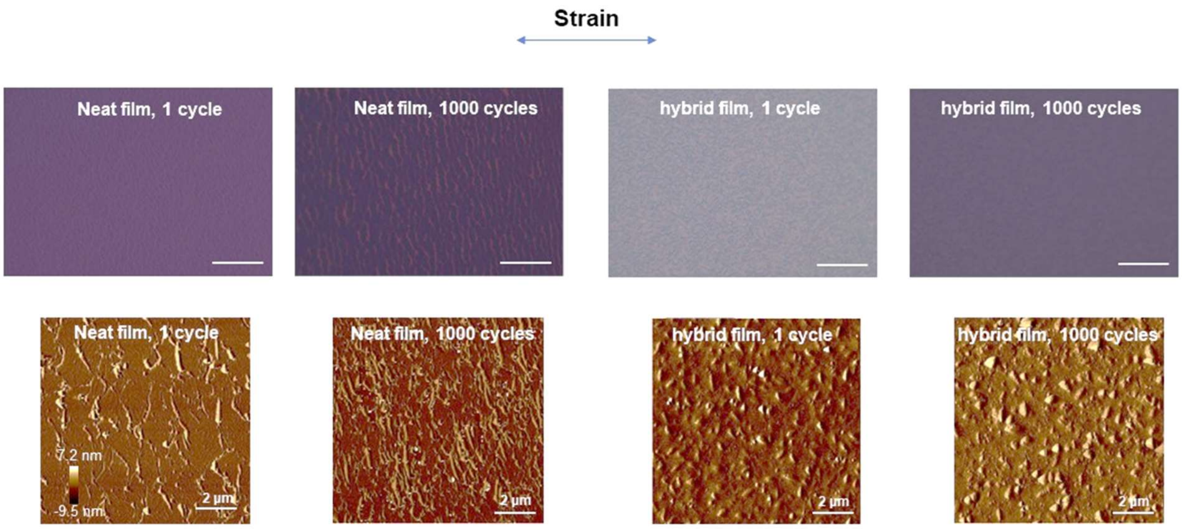

Supplementary Figure 17. Optical microscope and AFM phase images of the neat and hybrid films after multiple stretching-releasing cycles (up to 1000 cycles) at 25% strain.

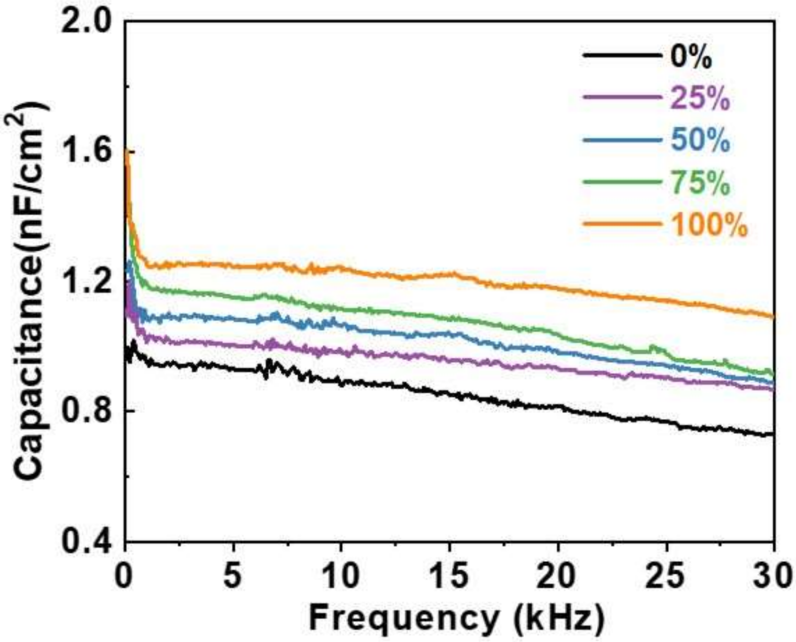

Supplementary Figure 18. Capacitance of the dielectric layer (initial thickness, d = 2000 nm) as a function of the frequency.

Supplementary Table 2. Device geometry and dielectric parameters for the fully stretchable transistor under different strains.

Stretching direction	Strain(%)	Channel length(μm)	Channel width(μm)	Dielectric Thickness (nm)	Capacitance (nF/cm^2)
Channel length	0	200	4000	2000±17	0.94
	25	250	3720	1690±26	1.02
	50	300	3440	1530±22	1.13
	75	350	3160	1410±18	1.22
	100	400	2880	1380±30	1.30
Channel width	0	200	4000	2000±17	0.94
	25	186	5000	1690±26	1.02
	50	172	6000	1530±22	1.13
	75	158	7000	1410±18	1.22
	100	144	8000	1380±30	1.30

Section 4. The photosensitivity evaluation of the n-type hybrid polymer films for the X-ray detectors.

1. Calculation of sensitivity

The sensitivity including the sensitivity per unit area (S_A) and sensitivity per unit volume(S_V), are defined as[7]:

$$S_A = \frac{I_{light} - I_{dark}}{DA};\ S_V = \frac{I_{light} - I_{dark}}{DV}$$

where I_{light} and I_{dark} represent the photocurrent and dark current, respectively. D is the dose rate of X-ray irradiation, and A or V represents the active area or volume of the organic semiconductor exposed under the X-ray irradiation, respectively.

Typically, the detection sensitivity can be derived from the slope of the fitting linear of the current density versus different dose rates. The expression is as follows[6]:

$$S_A = \partial J/\partial D;\ S_V = S_A/d$$

where J is the current density and d is the thickness of active layer. The current density is defined as[8-10]:

$$J = \frac{I}{WL}$$

where I is the current. W and L represent the width and length of the channel, respectively.

2. Calculation of photoconductive gain

The expression of gain (G) is depicted as follows[11]:

$$G = \frac{I_r}{I_t}$$

where I_r and I_t are the response current and theoretical current, respectively. The theoretical current is defined as[12]:

$$I_t = \phi nq$$

where Φ is the photon absorption rate, n is the number of the generated electron-hole pairs per absorbed photon, q is the elementary charge. It is noted that the total gain includes the photoconductive gain and impact ionization gain. To obtain the photoconductive gain, we consider $n = E_{ph}/\Delta$, $\Delta = 1.43 + 2E_g$, where E_{ph} is the X-ray photon energy and E_g is the energy gap of FIID-CF_3TVT ($E_g \approx 2.1$ eV). As for the impact ionization gain, n is determined by the ratio of E_{ph} to E_g.

$$\phi = \frac{\epsilon D m_s}{E_{ph}}$$

where ϵ is the fraction of absorbed photons, D is the dose rate, m_s is the sample mass.

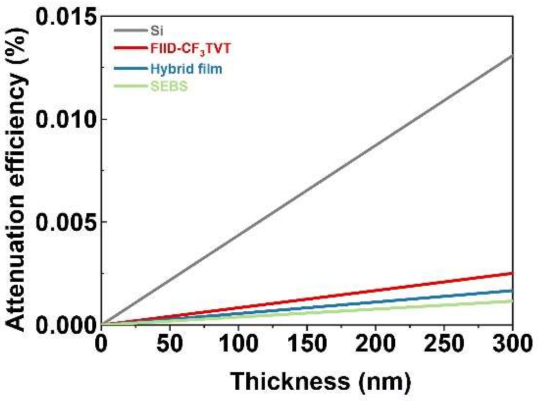

Supplementary Figure 19. Attenuation efficiency as a function of film thickness for the Si wafer, neat SEBS, neat FIID-CF_3TVT and hybrid semiconductor film when subjected to 20 keV X-ray photons.

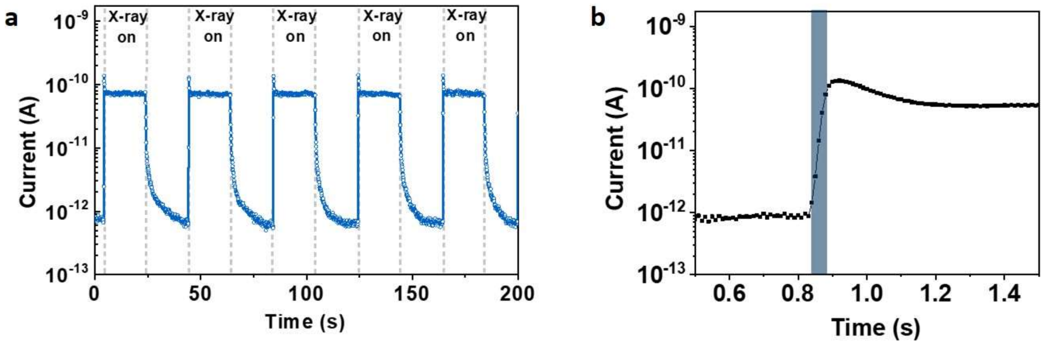

Supplementary Figure 20. Temporal response (a) and high-resolution temporal response (b) of the stretchable n-type hybrid semiconductor films at a X-ray dose rate of 3.5 $\mu Gy_{air}\ s^{-1}$ at $V_{DS} = 60V$ ($V_G = 0$ V).

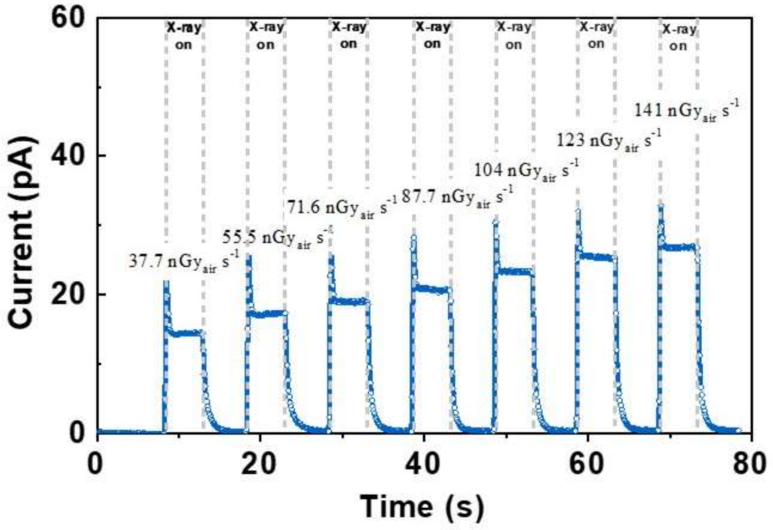

Supplementary Figure 21. Temporal response of the stretchable n-type hybrid semiconductor films under several different dose rates from 37.7 to 141 nGy_{air} s^{-1} at V_{DS} = 60V (V_G = 0 V).

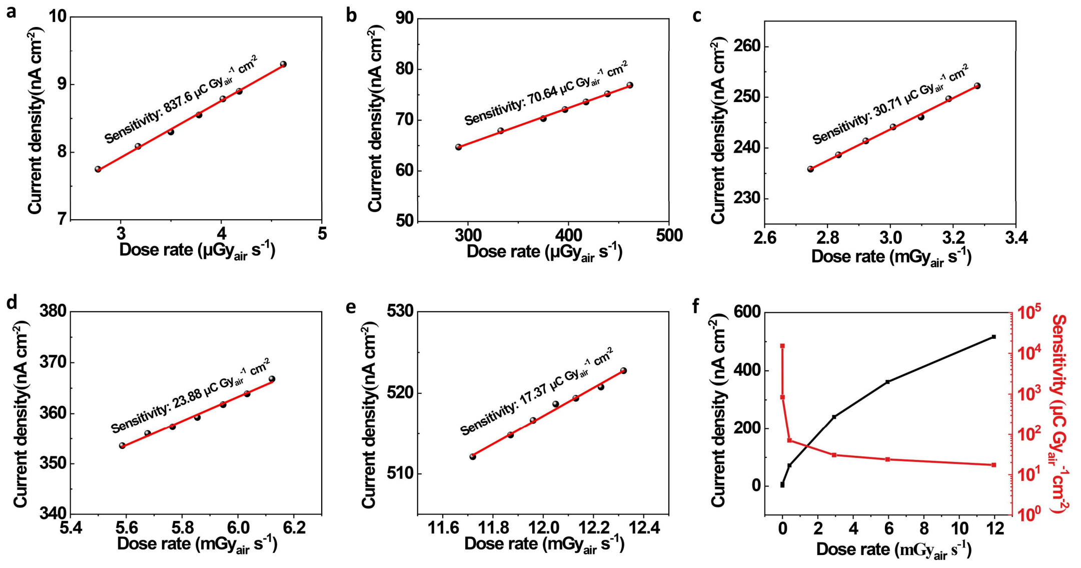

Supplementary Figure 22. Current density and photosensitivity as a function of X-ray dose rate for the hybrid polymer films at V_{DS} = 60V (V_G = 0 V).

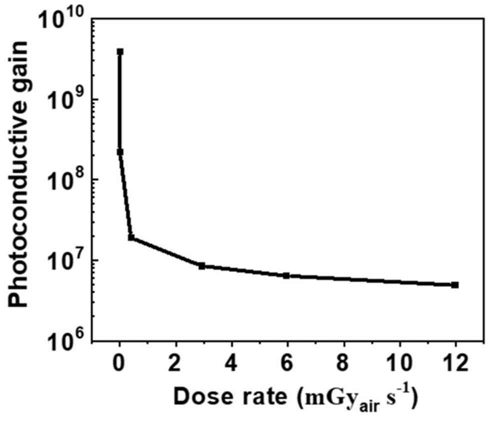

Supplementary Figure 23. Photoconductive gain as a function of X-ray dose rate for the hybrid polymer films at V_{DS} = 60V (V_G = 0 V).

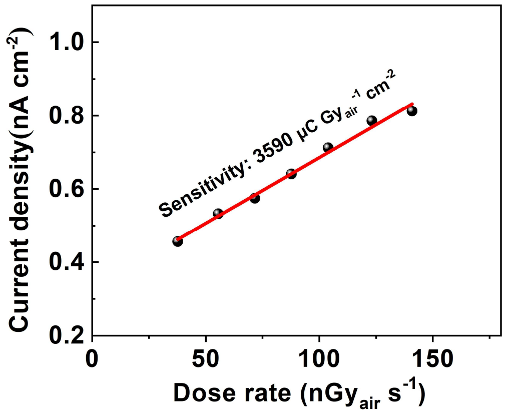

Supplementary Figure 24. Current density as a function of X-ray dose rate for the hybrid polymer films at V_{DS} = 10V (V_G = 0 V).

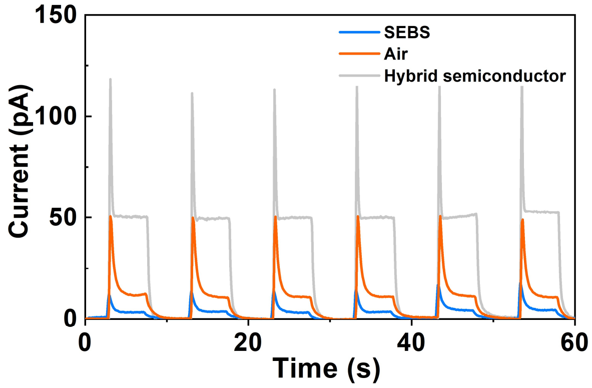

Supplementary Figure 25. Temporal response of the air, insulated SEBS and hybrid semiconductor film under 3.5 μGy_{air} s^{-1} dose rate at V_{DS} = 60V (V_G = 0 V).

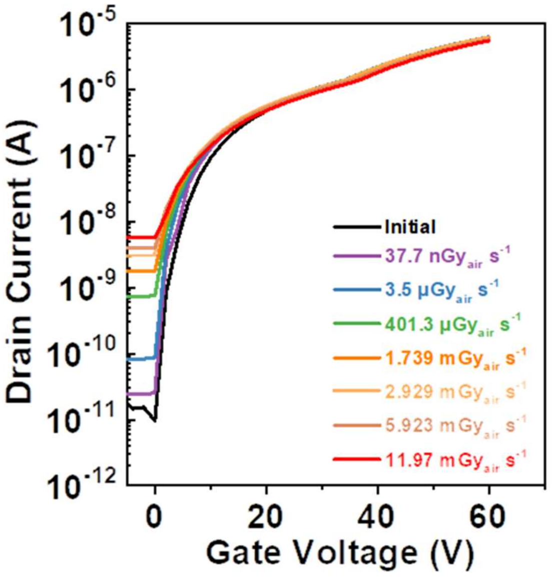

Supplementary Figure 26. Transfer characteristics of the STOFETs in the dark and under different dose rate from 37.7 nGy_{air} s^{-1} to 11.97 mGy_{air} s^{-1} at V_{DS} = 60V.

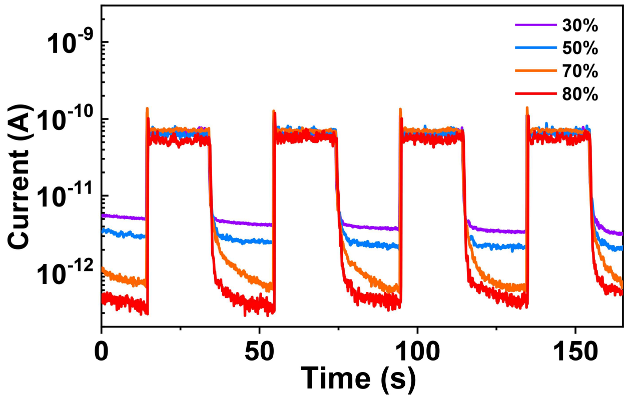

Supplementary Figure 27. Temporal response of the hybrid polymer films with different SEBS contents under 3.5 $\mu Gy_{air}\ s^{-1}$ dose rate at $V_{DS} = 60V$ ($V_G = 0$ V).

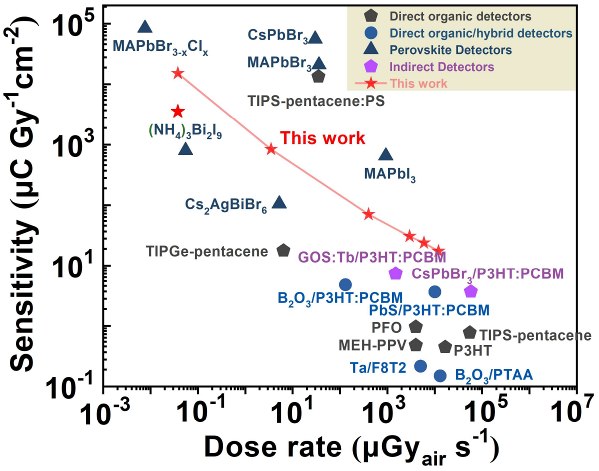

Supplementary Figure 28. Performance comparison including the sensitivity (S_A) and detection limit of current reported X-ray detectors with our prepared stretchable X-ray detectors in this work. The detailed conditions are concluded in Supplementary Table 3 (the single red pentagram, at $V_{DS} = 10V$).

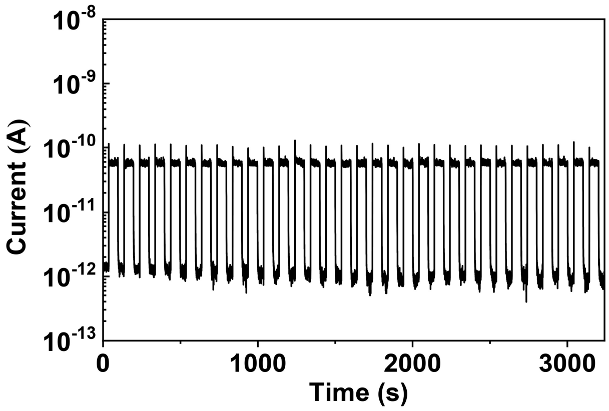

Supplementary Figure 29. Long-term response of the hybrid polymer film upon switching the X-ray irradiation on and off under 3.5 μGy_{air} s^{-1} dose rate at V_{DS} = 60V (V_G = 0 V).

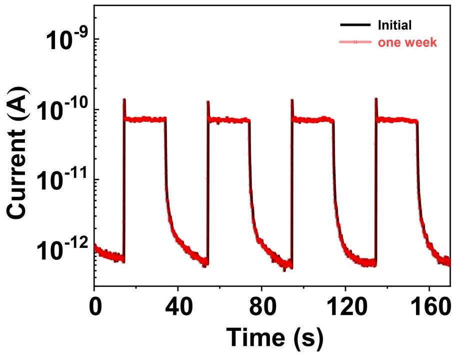

Supplementary Figure 30. Environmental stability of the initial hybrid polymer film and the polymer film stored for one week in air upon switching the X-ray irradiation on and off under 3.5 μGy_{air} s^{-1} dose rate at V_{DS} = 60V (V_G = 0 V).

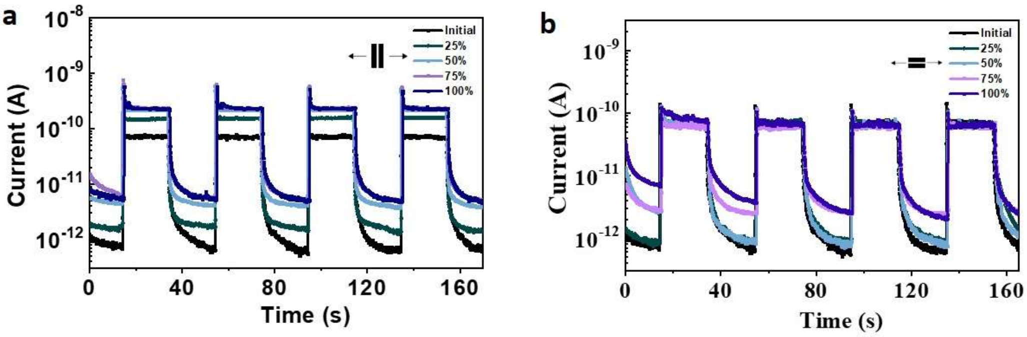

Supplementary Figure 31. Strain-tolerance behavior of the stretchable X-ray detectors with the hybrid polymer film under 3.5 μGy_{air} s^{-1} dose rate at V_{DS} = 60V (V_G = 0 V).

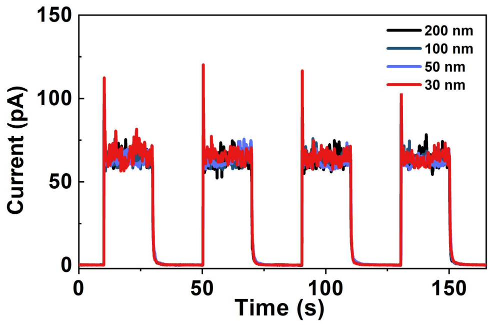

Supplementary Figure 32. Temporal response of the hybrid polymer semiconductor films with different thicknesses under 3.5 μGy_{air} s^{-1} dose rate at V_{DS} = 60V (V_G = 0 V).

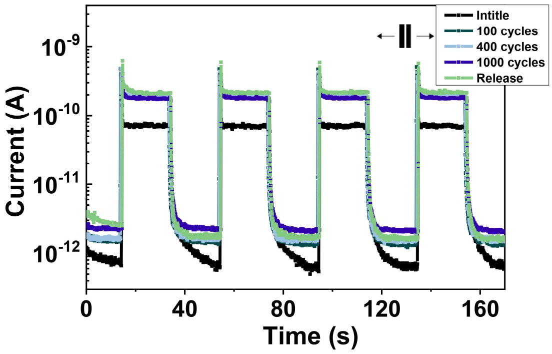

Supplementary Figure 33. Photoelectrical stability of the stretchable X-ray detectors with the hybrid polymer film under 3.5 μGy_{air} s^{-1} dose rate at V_{DS} = 60V (V_G = 0 V) after multiple stretching-releasing cycles (up to 1000 cycles) at 25% strain along the charge-transporting direction.

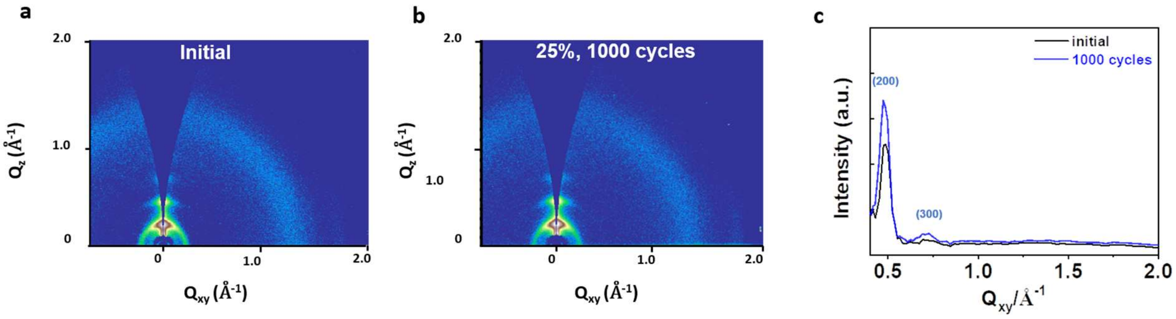

Supplementary Figure 34. GIWAXS images for the hybrid polymer film (a) and after 1000 stretch-release cycles (b) and line cuts along the Qxy axes (c).

Supplementary Table S3. Comparison in the performance of the prepared organic hybrid polymer with the previously reported X-ray photo-transistors.

The type of devices	Photoactive materials	Operate Voltage (V)	X-ray source	Minimum dose rate (mGy_{air} s^{-1})	S_A (μC Gy_{air}^{-1} cm^{-2})	S_V (μC Gy_{air}^{-1} cm^{-3})	Ref.
Indirect X-ray detectors	$Gd_2O_3:Eu^{3+}$:PPO:POPOP/FS0096	V_G= -5 to -45, V_{DS}= -45	Cu; 40 kV	--	--(R=1.457 μA/W)		13
Direct X-ray detector based on heterojunction phototransistors	PDPPBTT/$CsPbBrI_2$	V_G= -3, V_{DS}= -30	W; 20 keV	1×10^{-6}	~10^5	~10^9*	9
	P3HT/$CsPbBrI_2$	V_G= -60, V_{DS}= -30	W; 20 keV	1×10^{-6}	~10^5	~10^9*	
	$CsPbBr_3$/DPP-TT:SEBS	V_G= 0, V_{DS}= -60	Ag; 20 keV	7.9×10^{-5}	1.2×10^5*	1.2×10^9	14
Direct organic X-ray detectors	TIPGe-pentacene	V_G= -2, V_{DS}= -3	Mo; 35 kV	6.4×10^{-3}	18*	9.0×10^5	8
	TIPS-pentacene:PS	V_G= 0, V_{DS}= -20	Mo; 35 kV	3.5×10^{-2}	1.3×10^4	3.2×10^9	10
	TIPS-pentacene	V_G= -0.25, V_{DS}= -3	Mo; 35 kV	6.5	53*	5.3×10^6*	15
Direct organic X-ray detectors	FIID-CF_3TVT:SEBS	V_G= 0, V_{DS}= 60	Ag; 20 keV	3.77×10^{-5}	1.52×10^4	1.52×10^9	This work

*Values extracted by the device information reported in the referenced papers.

Supplementary Table S4. Comparison in the performance of the prepared organic hybrid polymer with the previously reported X-ray detectors using other materials and device structures.

The type of devices	Photoactive materials	Structure	Operate Voltage (V)	X-ray source (anode voltage/X-ray energy)	Minimum dose rate (mGy_{air} s^{-1})	S_A ($\mu C\ Gy_{air}^{-1}$ cm^{-2})	S_V ($\mu C\ Gy_{air}^{-1}$ cm^{-3})	Ref.
Perovskite detectors	$MAPbBr_{3-x}Cl_x$	Diode	-5	--; 8keV	7.60×10^{-6}	8.4×10^{4}	3.5×10^{5}	16
	$(NH_4)_3Bi_2I_9$	Resistance	10	Ag;50keV	5.50×10^{-5}	803	5.33×10^{4}	17
	$Cs_2AgBiBr_6$	Resistance	50	W; 50kV	5.2×10^{-3}	105	525	11
	$CsPbBr_3$	Diode	1.2	W; 50keV	3×10^{-2}	5.57×10^{4}	2.31×10^{6}	18
	$MAPbBr_3$	Diode	-1	Cu; --	3.6×10^{-2}	2.1×10^{4}	1.05×10^{5}	19
	$MAPbI_3$	Resistance	10	Cu;8keV	0.928	650*	2.2×10^{6}	20
Direct organic inorganic hybrid X-ray detectors	B_2O_3/P3HT:PCBM	Diode	-10	W; 50kV	0.13	4.79*	1.71×10^{3}	21
	Ta/F8T2	Diode	-50	Mo;17.5keV	5	0.217*	434	22
	B_2O_3/PTAA	Diode	-100	Mo;17.5keV	13	0.15*	75	
	PbS/P3HT:PCBM	Diode	-30	W; 40kV	10	3.66*	1219	23
Indirect organic inorganic hybrid X-ray detectors	GOS:Tb/P3HT:PCBM	Diode	-10	W; 70kV	1.5	7.35*	7300	24
	$CsPbBr_3$/P3HT:PCBM	Diode	-3	Cu; 40–80 kV	58.18	3.67	1837	25
	TIPS-pentacene	Resistance	0.1	Mo;17 keV	55	0.77*	7.7×10^{4}	26
	PFO	Diode	-50	Mo; 50keV	4	0.96*	480	27
	MEH-PPV	Diode	-10	Mo; 50keV	4	0.48*	240	
	P3HT	Diode	44	--	16.6	0.47*	154	28
Direct organic X-ray detectors	FIID-CF3TVT:SEBS	Transistor	V_G=0, V_{DS}=60	Ag; 20 keV	3.77×10^{-5}	1.52×10^{4}	1.52×10^{9}	This work
						359	3.59×10^{7}	

*Values extracted by the device information reported in the referenced papers.

Reference

1. Caddeo, C. et al. Hydrophilicity and Water Contact Angle on Methylammonium Lead Iodide. *Adv. Mater. Interfaces* **6**, 1801173 (2019).
2. Taylor, M., Urquhart, A.J., Zelzer, M., Davies, M.C. & Alexander, M.R. Picoliter Water Contact Angle Measurement on Polymers. *Langmuir* **23**, 6875-6878 (2007).
3. Xu, J. et al. Highly stretchable polymer semiconductor films through the nanoconfinement effect. *Science* **355**, 59-64 (2017).
4. Guo, Y.L., Yu, G. & Liu, Y.Q. Functional Organic Field-Effect Transistors. *Adv. Mater.* **22**, 4427-4447 (2010).
5. Xu, J. et al. Multi-scale ordering in highly stretchable polymer semiconducting films. *Nat. Mater.* **18**, 594-602 (2019).
6. Kong, D. et al. Capacitance Characterization of Elastomeric Dielectrics for Applications in Intrinsically Stretchable Thin Film Transistors. *Adv. Funct. Mater.* **26**, 4680-4686 (2016).
7. Chen, M., Wang, C. & Hu, W. Organic photoelectric materials for X-ray and gamma ray detection: mechanism, material preparation and application. *J. Mater. Chem. C* (2021).
8. Ciavatti, A. et al. Boosting Direct X-Ray Detection in Organic Thin Films by Small Molecules Tailoring. *Adv. Funct. Mater.* **29**, 1806119 (2018).
9. Gao, Y. et al. Ultrathin and Ultrasensitive Direct X-ray Detector Based on Heterojunction Phototransistors. *Adv. Mater.* **33**, e2101717 (2021).
10. Temiño, I. et al. Morphology and mobility as tools to control and unprecedentedly enhance X-ray sensitivity in organic thin-films. *Nat. Commun.* **11**, 2136 (2020).
11. Pan, W. et al. Cs2AgBiBr6 single-crystal X-ray detectors with a low detection limit. *Nat. Photonics* **11**, 726-732 (2017).
12. Fraboni, B. et al. Organic Semiconducting Single Crystals as Next Generation of Low-Cost, Room-Temperature Electrical X-ray Detectors. *Adv. Mater.* **24**, 2289-2293 (2012).
13. Oliveira, J. et al. Indirect X-ray Detectors Based on Inkjet-Printed Photodetectors with a Screen-Printed Scintillator Layer. *ACS Appl. Mater. Interfaces* **10**, 12904-12912 (2018).
14. Liu, K. et al. Ultrahigh-Performance Optoelectronic Skin Based on Intrinsically Stretchable Perovskite-Polymer Heterojunction Transistors. *Adv. Mater.* **0**, 2107304 (2021).
15. Lai, S. et al. A Highly Sensitive, Direct X-Ray Detector Based on a Low-Voltage Organic Field-Effect Transistor. *Adv. Electron. Mater.* **3**, 1600409 (2017).
16. Wei, H. et al. Dopant compensation in alloyed CH3NH3PbBr3−xClx perovskite single crystals for gamma-ray spectroscopy. *Nat. Materials,* **16**, 826-833 (2017).
17. Zhuang, R. et al. Highly sensitive X-ray detector made of layered perovskite-like (NH4)3Bi2I9 single crystal with anisotropic response. *Nat. Photonics* **13**, 602-608 (2019).
18. Pan, W. et al. Hot-Pressed CsPbBr3 Quasi-Monocrystalline Film for Sensitive Direct X-ray Detection. *Adv. Mater.* **31**, 1904405 (2019).
19. Wei, W. et al. Monolithic integration of hybrid perovskite single crystals with heterogenous substrate for highly sensitive X-ray imaging. *Nat. Photonics* **11**, 315-321 (2017).
20. Yakunin, S. et al. Detection of gamma photons using solution-grown single crystals of hybrid lead halide perovskites. *Nat. Photonics* **10**, 585-589 (2016).
21. Thirimanne, H.M. et al. High sensitivity organic inorganic hybrid X-ray detectors with direct transduction and broadband response. *Nat. Commun.* **9**, 2926 (2018).

22. Mills, C.A. et al. Enhanced x-ray detection sensitivity in semiconducting polymer diodes containing metallic nanoparticles. *J. Phys. D: Appl. Phys.* **46**, 275102 (2013).
23. Ankah, G.N. et al. PbS quantum dot based hybrid-organic photodetectors for X-ray sensing. *Org. Electron.* **33**, 201-206 (2016).
24. Büchele, P. et al. X-ray imaging with scintillator-sensitized hybrid organic photodetectors. *Nat. Photonics* **9**, 843-848 (2015).
25. Xiang, L. et al. X-ray Sensitive hybrid organic photodetectors with embedded CsPbBr3 perovskite quantum dots. *Org. Electron.* **98**, 106306 (2021).
26. Basiricò, L. et al. Direct X-ray photoconversion in flexible organic thin film devices operated below 1 V. *Nat. Commun.* **7**, 13063 (2016).
27. Boroumand, F.A. et al. Direct x-ray detection with conjugated polymer devices. *Appl. Phys. Lett.* **91**, 033509 (2007).
28. Valitova, I., Hupman, A., Hill, I.G. & Syme, A. Poly(3-hexylthiophene-2,5-diyl) based diodes for ionizing radiation dosimetry applications. *Org. Electron.* **88**, 105981 (2021).
